# Interpretable AI-driven multi-objective risk prediction in heart failure patients with thyroid dysfunction

**DOI:** 10.3389/fdgth.2025.1583399

**Published:** 2025-05-12

**Authors:** Massimo Iacoviello, Vito Santamato, Alessandro Pagano, Agostino Marengo

**Affiliations:** ^1^ Department of Clinical and Experimental Medicine, University of Foggia, Foggia, Italy; ^2^ Department of Agriculture, Food, Natural Resources and Engineering Sciences, University of Foggia, Foggia, Italy; ^3^ Department of Computer Science, University of Bari Aldo Moro, Bari, Italy

**Keywords:** artificial intelligence, machine learning, heart failure, thyroid dysfunction, risk stratification, explainable AI

## Abstract

**Introduction:**

Heart Failure (HF) complicated by thyroid dysfunction presents a complex clinical challenge, demanding more advanced risk stratification tools. In this study, we propose an AI-driven machine learning (ML) approach to predict mortality and hospitalization risk in HF patients with coexisting thyroid disorders.

**Methods:**

Using a retrospective cohort of 762 HF patients (including euthyroid, hypothyroid, hyperthyroid, and low T3 syndrome cases), we developed and optimized several ML models—including Random Forest, Gradient Boosting, Support Vector Machines, and others—to identify high-risk individuals.

**Results:**

The best-performing model, a Random Forest classifier, achieved robust predictive accuracy for both 1-year mortality and HF-related hospitalization (area under the ROC curve ∼0.80 for each). We further employed model interpretability techniques (Local Interpretable Model-agnostic Explanations, LIME) to elucidate key predictors of risk at the individual level. This interpretability revealed that factors such as atrial fibrillation, absence of cardiac resynchronization therapy, amiodarone use, and abnormal thyroid-stimulating hormone (TSH) levels strongly influenced model predictions, providing clinicians with transparent insights into each prediction. Additionally, a multi-objective risk stratification analysis across thyroid status subgroups highlighted that patients with hypothyroidism and low T3 syndrome are particularly vulnerable under high-risk conditions, indicating a need for closer monitoring and tailored interventions in these groups.

**Discussion:**

In summary, our study demonstrates an innovative AI methodology for medical risk prediction: interpretable ML models can accurately stratify mortality and hospitalization risk in HF patients with thyroid dysfunction, offering a novel tool for personalized medicine. These findings suggest that integrating explainable AI into clinical workflows can improve prognostic precision and inform targeted management, though prospective validation is warranted to confirm realworld applicability.

## 1 Introduction

Heart Failure (HF) is one of the leading causes of morbidity and mortality globally, imposing a significant burden on healthcare systems and the quality of life of patients. Concurrently, thyroid dysfunctions, particularly hypothyroidism, have been associated with worsening clinical outcomes in patients with HF, adversely affecting prognosis. Recent studies underscore that subclinical hypothyroidism (SH) significantly raises the risk of cardiovascular mortality in HF patients, emphasizing the need for precise monitoring and intervention strategies (1). Optimal ranges of thyroid-stimulating hormone (TSH) and free thyroxine (FT4) levels are linked to reduced mortality risks, suggesting that both high and low extremes can worsen HF outcomes (2). Previous studies have demonstrated that hypothyroidism can negatively impact cardiac function and increase the risk of developing HF. Recent meta-analyses have confirmed that subclinical hypothyroidism is associated with an increased risk of all-cause mortality and hospitalization in patients with HF, highlighting the importance of thyroid evaluation in this population (3). However, the relationship between hypothyroidism, HF, and mortality remains complex and multifactorial, requiring further exploration for optimal patient management.

The complexity of clinical management of this patient cohort underscores the need for advanced tools for accurate and personalized risk assessment. Machine learning (ML) has shown revolutionary capabilities in the medical field, particularly in predictive medicine, where complex models such as XGBoost, Random Forest, and LightGBM have managed large volumes of clinical data and identified complex patterns not immediately apparent to human analysis (4). Recent advancements, such as the use of SF-IIAdaboost algorithms integrating IoT and AI, have achieved high predictive accuracy in cardiovascular contexts, underscoring the potential for enhanced prognostic precision (5). The use of advanced ML algorithms has enabled the identification of clinical and biochemical features that predict mortality risk, examining how these interact with each other and with the patient's baseline condition. Such models have been shown to improve risk stratification and treatment personalization in patients with HF, including those in a hypothyroid state (6). In patients with HF, ML analysis has identified prognostic phenotypes, facilitating the application of precision medicine. This approach is particularly relevant for hypothyroid patients, who present a unique disease dynamic compared to patients with overt thyroid dysfunction (7).

This work aims to explore the application of ML in estimating the mortality risk in hypothyroid patients suffering from HF, with a particular emphasis on the analysis of age and TSH levels as prognostic factors. Through the analysis of a large cohort of cardiac patients stratified by thyroid conditions, this study aims to develop ML models that provide accurate estimates for two main targets: mortality and hospitalization in this specific population. Our goal is twofold: on one hand, to contribute to the scientific literature by offering insights into the underlying mechanisms of the association between thyroid conditions and HF; on the other hand, to provide healthcare providers with an innovative tool for improving risk stratification and personalizing therapeutic strategies.

The core of this work involves the presentation of the research methods used to develop the ML models, including feature selection, model training, and validation. Finally, the results are analyzed in detail, highlighting how various factors contribute to predicting the risk of mortality and hospitalization in patients with HF and how these models can be employed in clinical practice to support more informed therapeutic decisions.

The use of ML in predicting mortality risk in patients with HF could mark a significant advancement in managing this complex intersection of conditions. This study aims to explore such potential, opening new frontiers in cardiovascular and endocrinological research. By highlighting these computational underpinnings, the manuscript extends the theoretical understanding of explainable AI in clinical contexts and bridges the gap between algorithmic transparency and medical applicability. The article begins in Section 2 with a comprehensive background, offering an overview of related studies and showcasing the unique benefits and objectives of this research. In Section 3, the methodology is detailed, guiding readers through the study's innovative approach. Section 4 dives into a discussion of the primary findings, spotlighting key results and their implications. Finally, the conclusion ties everything together, underscoring the study's contributions and future directions.

## 2 Background

The growing awareness of the negative impact of hypothyroidism on patients with HF underscores the need for comprehensive risk assessment and personalized management strategies. Studies have shown that hypothyroidism, including its subclinical form, is prevalent among HF patients and significantly contributes to an increased risk of mortality, hospitalization, and deterioration of cardiac function. Amiodarone, a commonly used antiarrhythmic drug, has been identified as a determining factor in the onset of hypothyroidism in this population (8). Research highlights the importance of monitoring TSH levels as a key indicator of thyroid function in these patients. It has been demonstrated that correcting thyroid hormone deficiency, indicated by elevated TSH levels, leads to improvements in cardiac function while simultaneously reducing the risk of hospitalization and mortality. Conversely, worsening thyroid function, characterized by rising TSH levels, is associated with a decline in cardiac function and adverse outcomes (9, 10). Beyond traditional risk markers, the role of N-terminal pro-B-type natriuretic peptide (NT-proBNP) has emerged as a significant prognostic factor in patients with suspected HF. Even in the absence of echocardiographic evidence of HF, elevated NT-proBNP levels, combined with factors such as advanced age, male sex, chronic kidney disease (CKD), chronic obstructive pulmonary disease (COPD), and dementia, have been associated with higher mortality (11). These findings highlight the complex interaction between HF and thyroid dysfunction, suggesting a need for more sophisticated approaches for accurate risk stratification and timely interventions.

The emergence of Machine Learning (ML) algorithms such as XGBoost, Random Forest, and LightGBM offers a promising avenue forward. These ML algorithms have demonstrated their ability to discern complex prognostic patterns and improve treatment personalization in various healthcare contexts, including predicting acute kidney injury (AKI) following percutaneous coronary intervention (PCI) in patients with acute coronary syndrome (ACS) (12). In HF, recent studies indicate that ML models enhance predictive accuracy for mortality and readmission by integrating comprehensive clinical data and managing issues like data imbalance and incompleteness (13). Advanced deep learning techniques, such as multi-head self-attention, further improve model performance, particularly in handling complex and diverse datasets common in HF populations (14). Applying ML algorithms in this context may improve the precision of risk assessment and support more personalized management of patients with HF and hypothyroidism, although prospective validation is still required. By harnessing the power of these algorithms, we could develop predictive models capable of accurately identifying high-risk individuals for adverse outcomes, allowing for targeted interventions and improved patient outcomes. Additionally, the integration of variables such as age and TSH levels into ML models could provide further insights into the delicate balance between cardiac and thyroid function. By incorporating these factors, the resulting models may achieve higher predictive accuracy, guiding clinical decisions and leading to personalized treatment strategies.

### 2.1 Related studies and benefits

Recent scientific literature highlights the effectiveness of ML in predicting complex clinical outcomes, such as mortality and hospitalization, especially in patients with endocrine and cardiovascular comorbidities. Some studies have explored the use of ML to analyze autoimmune and endocrine diseases, revealing the significant role that conditions like diabetes and thyroid disorders play in elevating mortality rates (15). Similarly, other studies have applied ML to diagnose forms of secondary hypertension, showing how abnormal TSH levels can influence cardiovascular risk (16). Additionally, models have emerged linking diabetes and hypothyroidism with increased mortality in COVID-19 patients requiring hospitalization (17), while other research has developed algorithms to predict atrial fibrillation associated with thyrotoxicosis, emphasizing the importance of thyroid profiles in heart disease (18). Further investigations into the connection between subclinical hypothyroidism and cardiovascular diseases have also examined the potential for accurately predicting mortality and hospitalization in patients with HF (19, 20). ML models that incorporate social determinants of health have also shown promise in predicting in-hospital mortality for HF patients, illustrating the benefits of integrating clinical and social factors to improve outcomes in complex cardiovascular cases (21). Efforts to enhance cardiovascular risk predictions by integrating factors such as diabetes and thyroid health have further refined risk stratification models (22). Additionally, there is promising research on ML frameworks that predict postprocedural outcomes in interventional radiology using random forest models, offering insight into complications, mortality, and length of stay (23). However, these studies often treat thyroid dysfunctions as one of many risk variables, without fully exploring their specific impact on patients with cardiovascular conditions.

This study stands out by providing a detailed, targeted analysis of the influence of thyroid conditions on clinical outcomes through an innovative ML approach. Unlike previous studies, this work focuses specifically on the impact of thyroid dysfunctions, making each prediction more precise and tailored to clinical management. Additionally, by using Local Interpretable Model-agnostic Explanations (LIME), predictions are both transparent and individualized, allowing clinicians to clearly see how each clinical variable contributes to the risk of mortality or hospitalization for each patient, thereby supporting more informed and personalized decision-making.

The ML analysis also extends to specific patient subgroups, such as euthyroid and hypothyroid patients, making this study uniquely comprehensive compared to existing literature. Through advanced predictive modeling, the study has identified the absence of Cardiac Resynchronization Therapy (CRT) as a critical risk factor for mortality in patients with thyroid dysfunctions, suggesting that targeted interventions could improve patient prognosis. Another key finding is the association between low TSH levels and reduced hospitalization risk in euthyroid patients, introducing new parameters to monitor even in the absence of overt hypothyroidism or hyperthyroidism. Finally, ML has enabled the identification of an increased mortality risk associated with Amiodarone use in patients with LT3, offering practical insights for optimizing therapeutic decisions in cardiology.

In summary, this study not only enriches scientific knowledge but also serves as an innovative pillar for precision medicine in managing patients with thyroid and cardiovascular comorbidities. The advanced use of ML enables more accurate and personalized predictions, thus transforming the quality of clinical care.

### 2.2 Patient selection

In this study, we examined a cohort of 762 patients to assess significant clinical outcomes such as HF hospitalization and mortality over the follow-up period. The patients were monitored for durations ranging from less than a month to almost 12.7 years, with an average follow-up period of approximately 4.5 years (9).

The selection of participants was meticulously conducted to include only those subjects for whom complete data were available regarding arrival date, follow-up date, age, sex, and key clinical events such as mortality and HF hospitalization. No patient was excluded due to a lack of essential data, thus maintaining the integrity of the cohort.

From a demographic perspective, the average age of participants at the time of arrival was 63.5 years, ranging from 14 to 89 years. Males constituted 78% of the cohort, demonstrating a prevalence of this gender. This sex imbalance reflects the characteristics of the referred population but may also introduce gender-related bias, particularly relevant given the higher prevalence of thyroid dysfunction in females. Regarding clinical outcomes, about 30% of the patients died, and 22% experienced at least one episode of HF hospitalization during the follow-up period. All consecutive outpatients with CHF referred to the HF Unit of the University Policlinic Hospital of Bari from January 2006 to December 2016 were retrospectively evaluated. All the evaluations with patients in stable clinical conditions from at least 30 days and in conventional medical and electrical therapy from at least 3 months were considered. The adoption of well-defined inclusion criteria minimized potential biases arising from incomplete data and enhanced the representativeness and generalizability of the results. For patients who developed thyroid dysfunction after their initial evaluation, the clinical timepoint corresponding to the diagnosis of hypothyroidism, hyperthyroidism, or low-T3 syndrome was considered as the analytical baseline (9). This allowed for consistent classification of thyroid status and ensured that risk predictions were anchored to the relevant endocrine condition.

## 3 Materials and methods

The study is based on a dataset of 762 patients and employs ML techniques implemented in Python to build predictive models that estimate the risks of mortality and hospitalization. The main objective is to analyze the influence of various clinical characteristics, including thyroid variables, on these outcomes.

**Figure 1:**
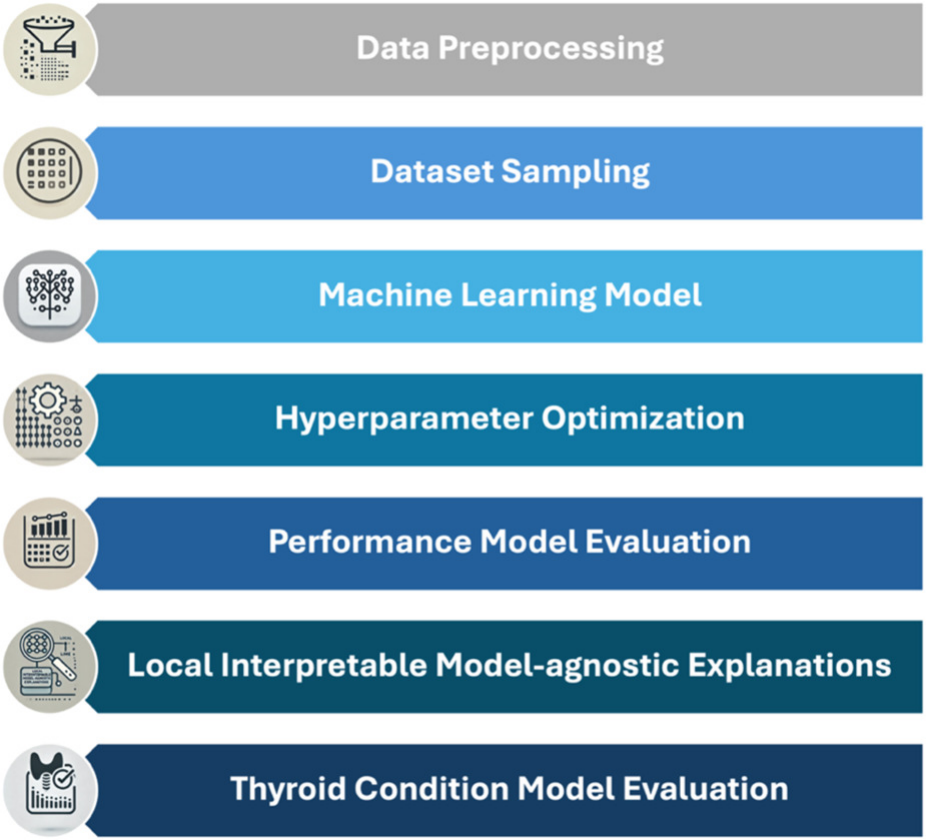
Workflow diagram for data collection and ML model training.

The analyses were conducted using Orange Data Mining software version 3.36.2 on an Apple M1 Pro system equipped with 16 GB of RAM and 1 TB of storage, operating on macOS Sonoma 14.2.1. This setup, combined with the use of advanced ML techniques, ensured the efficiency and reproducibility of our analyses. The importance of such ML methodologies in extracting meaningful insights and predictive models from complex datasets has been previously highlighted and validated in similar studies in the field of health performance assessment, such as efficiency and mobility (24–27) and for predicting neurodevelopmental disorders in children (28). The methodological phases of the study, illustrated in Figure 1, were developed in a Python environment, highlighting the key steps of the analysis.

The methodological workflow, illustrated in Figure 1, follows a multi-step approach organized into key phases:

1. Data preprocessing and handling of missing data: Missing data is managed through model-based methods that leverage relationships among variables to estimate missing values, preserving the original distribution and minimizing potential bias.
2. Dataset sampling: To assess model robustness, the dataset is split into a training set and a test set, allowing for rigorous validation of predictive performance.
3. Selection of ML models: Various ML algorithms are tested, including Random Forest, Gradient Boosting, Naive Bayes, Support Vector Machine, K-Nearest Neighbors, Neural Networks, Decision Trees, AdaBoost, Stochastic Gradient Descent, and Logistic Regression.
4. Internal Validation and Hyperparameter Optimization: Techniques such as grid search and cross-validation are employed to optimize hyperparameters, ensuring that model performance generalizes and is not limited to the training set alone.
5. Performance Model Evaluation: An evaluation function is created to automate model assessment on the test data, calculating metrics such as area under the ROC curve (AUC), accuracy, F1-score, precision, recall, and MCC to facilitate model comparison.
6. Model interpretation with LIME: To interpret predictions, LIME is used, highlighting the contribution of each variable to the final prediction and providing visual representations accessible to a non-technical audience.
7. Evaluation of models on different thyroid conditions: Models are evaluated on both the entire dataset and subgroups based on thyroid conditions (Euthyroidism, Hypothyroidism, Hyperthyroidism, and Low T3 Syndrome). This approach allows exploration of how model performance varies according to different thyroid conditions.

In summary, the study adopts a ML approach to develop and validate predictive models for mortality and hospitalization risks in cardiology and endocrinology patients. The workflow incorporates multiple phases, from data preprocessing to model interpretation, with particular attention to the influence of thyroid variables.

### 3.1 Dataset

Initially, we collected a broad set of clinical data, including both numerical and categorical variables ranging from demographic to biochemical parameters (29). In accordance with the study conducted by Iacoviello et al. in 2020, for each patient, the baseline evaluation was conducted during the first recorded medical visit. At this stage, a comprehensive medical history, physical examination, 12-lead ECG, mono- and two-dimensional echocardiographic evaluation, and blood samples were collected. For patients who subsequently developed thyroid disorders, the evaluation corresponding to the diagnosis of hypothyroidism, hyperthyroidism, or low T3 syndrome (LT3) was considered as the baseline. During the medical visit, the presence of ischemic cardiomyopathy, arterial hypertension, atrial fibrillation, and diabetes mellitus was carefully documented, along with any previous thyroid disease diagnosis. Data on HF therapy and any prior or ongoing treatment with amiodarone were also gathered. Additionally, information regarding the thyroid disease diagnosis was recorded. The 12-lead ECG was used to assess heart rhythm and rate. Echocardiographic recordings were obtained using a phased-array echo-Doppler system (Sonos 5500, Philips, Netherlands; from September 2008 onward, Vivid 7, GE, Wisconsin, USA) to estimate the left ventricular ejection fraction (LVEF) using the Simpson method. At baseline, levels of sodium (mEq/L), serum creatinine concentrations (mg/dl), and hemoglobin (g/dl) were measured. The glomerular filtration rate (GFR, ml/min) was calculated using the EPI formula (30). Additionally, amino-terminal brain natriuretic peptide (NT-proBNP, Dade Behring, Eschborn, Germany), free T3 (fT3), free thyroxine (fT4), and TSH levels were measured through immunoassays, using the reference ranges provided by the kit manufacturers (Advia Centaur, Bayer HealthCare, Diagnostics Division, Tarrytown, NY, US until 2011, and subsequently Dimension Vista, Siemens Healthcare Diagnostics, Erlangen, Germany). The resulting dataset with the selected variables is shown in Table 1.

**Table 1:** Overview of variables in the dataset.

Model variable	Variable name	Description	Type variable
Target	Mortality	Patient mortality event (1: Yes, 0: No)	Categorical
	HF hospitalization	Patient hospitalization (1: Yes, 0: No)	
FEATURE	Male gender	Patient's gender (1: male, 0: female)	
	Ischemic cardiomiopaty	Presence of ischemic cardiomyopathy (1: present, 0: absent)	
	Diabetes	Diabetes diagnosis (1: Diabetic, 0: non-diabetic)	
	ACEi/ARBs	Use of ACE inhibitors or ARBs (1: Use, 0: no use)	
	Beta-blockers	Use of beta-blockers (1: Use, 0: no use)	
	Diuretics	Use of diuretics (1: Use, 0: no use)	
	Aldosterone antagonists	Use of aldosterone antagonists (1: Use, 0: no use)	
	Amiodarone	Use of amiodarone (1: Use, 0: no use)	
	ICD	Implantable defibrillator (1: Present, 0: absent)	
	CRT	Cardiac resynchronization therapy (1: present, 0: absent)	
	NYHA class	NYHA functional class (1, 2, 3)	
	Atrial fibrillation	Presence of atrial fibrillation (1: present, 0: absent)	
	Age	Patient's age (years)	Numerical
	BMI	Body mass index (kg/m²)	
	Systolic arterial pressure	Systolic blood pressure (mmHg)	
	LVEF	Calculated ejection fraction (percentage)	
	GFR-EPI	Estimated glomerular filtration rate (ml/min/1.73 m²)	
	Natremia	Blood sodium concentration (mmol/L)	
	NT-proBNP	NT-proBNP levels in blood (pg/ml)	
	FT3	Free triiodothyronine levels (pmol/L)	
	FT4	Free thyroxine levels (pmol/L)	
	TSH	TSH levels (mU/L)	

The table provides a comprehensive description of the variables used to feed our ML model for predicting two key clinical outcomes: mortality and hospitalization. The variables are organized into two main categories, namely *Target*, which includes the clinical outcomes of interest, and *Feature*, which comprises the relevant clinical and demographic factors selected to optimize the predictive accuracy of the model.

In the *Target* category, there are two variables, “Mortality” and “Hospitalization,” which respectively indicate the occurrence of patient mortality and hospitalization. Each is coded as a categorical variable, with the value 1 representing the occurrence of the event and the value 0 indicating its absence. These targets serve as the dependent variables of the model, which is trained to identify and classify the risks associated with each outcome.

The *Features* include a range of demographic and clinical variables, carefully selected to identify significant correlations and enhance the model's predictive capabilities. Among the demographic characteristics, *MALE GENDER* indicates the patient's gender, with 1 for male and 0 for female, an important attribute as gender can influence HF prognosis. The patient's age is represented by the continuous numeric variable *AGE*, allowing the model to capture risk variations associated with advanced age. The body mass index *BMI*, expressed in kg/m², is also included as a general health indicator, potentially associated with overall cardiovascular risk.

The clinical variable set consists of critical diagnostic information, such as the presence of ischemic cardiomyopathy, described by the variable *ISCHEMIC CARDIOMYOPATHY*, and diabetes diagnosis, represented by the *DIABETES* variable. Both are binary variables distinguishing between patients with and without these conditions, each known to negatively impact the progression of HF. Other clinical variables include pharmacological treatments followed by the patients, such as the use of ACE inhibitors or angiotensin receptor blockers ACEinhibitor/ANGIOTENSIN II RECEPTOR BLOCKERS (*ACEi/ARBs)*, *BETA-BLOCKERS*, *DIURETICS*, and *MINERALCORTICOID RECEPTOR ANTAGONISTS*. These medications, coded as 1 for use and 0 for non-use, play a crucial role in managing symptoms and preventing cardiovascular complications. The use of *AMIODARONE*, an antiarrhythmic drug, is similarly included as a binary variable, as it is relevant for patients with severe arrhythmias. *ATRIAL FIBRILLATION* is a key clinical feature indicating the presence of atrial fibrillation, coded as 1 for present and 0 for absent. This variable is essential for HF patients, as atrial fibrillation can exacerbate symptoms and increase the risk of adverse events.

The model also incorporates instrumental characteristics, such as the presence of an implantable cardioverter-defibrillator *ICD* and cardiac resynchronization therapy *CRT,* both coded to indicate the presence or absence of the device, respectively with 1 and 0. The patient's *NYHA* functional class, categorized with values from 1 to 3, is another critical clinical parameter, as it reflects the severity of HF symptoms and helps predict the risk of adverse events.

The dataset further includes a series of relevant physiological and biochemical parameters, such as systolic blood pressure, measured in mmHg, and the calculated ejection fraction (*LVEF)*, expressed as a percentage, which represent the level of blood pressure and the heart's contractile capacity, respectively. Renal function is evaluated through the estimated glomerular filtration rate by EPI formula (*GFR-EPI*), measured in ml/min/1.73 m², while blood sodium concentration (*NATREMIA*) provides insights into electrolyte balance and fluid regulation, both relevant to cardiovascular function. Amino-terminal Brain Natriuretic Peptide (*NT-proBNP)*, a biomarker of HF severity, is also included and measured in pg/ml to quantify the condition's severity.

The dataset is completed by the levels of the thyroid hormones *FT3* and *FT4*, along with *TSH*, which offer valuable information about the patient's thyroid function. These variables are particularly significant for patients with thyroid dysfunction, given their potential impact on outcomes in HF.

This set of variables forms a robust and multidimensional data foundation essential for training ML models. Through this wide array of clinical and demographic features, the ML model can process complex details and identify significant patterns, thereby providing valuable support in predicting clinical risks and personalizing therapies for patients with HF and associated comorbidities.

### 3.2 Preprocessing and data sampling

These data were meticulously cleaned to eliminate anomalies and missing values, thereby ensuring the integrity of the dataset used for model training. The handling of 0.2% missing data was performed using the *model-based imputer* with a simple tree model, through Orange (version 3.36.2), a data mining software built on open-source Python libraries for scientific computing, such as NumPy and SciPy. The *Impute* widget was used for this purpose, allowing the construction of models to predict missing values based on the available data in other variables. With the integration of advanced Python libraries, Orange provides a powerful interface for imputation and scientific calculations, enabling accurate estimation of missing values with a simple decision tree while preserving dataset integrity, even with a low percentage of missing data.

Mathematically, the imputation process can be represented as follows: each missing value $X_{i}$ is estimated using other observed variables $X_{-i}$ through a function *f* derived from a simple decision tree, as shown in (Equation 1):

(1) $$\hat{X_{i}}=f(X_{-i})$$

Where $\hat{X_{i}}$ denotes the imputed value for the variable $X_{i}$, $X_{-i}$ represents the set of all other observed variables used as predictors, and *f* is the function constructed by the decision tree to predict the missing values.

For continuous variables, this function imputes missing values as the mean of known values within the relevant leaf node, as described in (Equation 2):

(2) $$\hat{X_{i}}=\frac{1}{n}\;\underset{\;j\in leaf}{\sum }X_{j}$$

where *n* is the number of samples in the same leaf node and $X_{j}\;$represents each known value of $X_{i}$ within that node. The summation $\underset{\;j\in leaf}{\sum }X_{j}$ calculates the total of known values for $X_{i}\;$in the node, with the division by *n* yielding the mean.

Equations 1, 2 together provide the general method for accurately filling in missing values, preserving dataset integrity for effective model training.

The dataset was divided into a training set (70%) and a validation set (30%), using this split to minimize the risk of overfitting and to verify the model's ability to generalize to unseen data. This split was done in Python using the *train_test_split* command of the *sklearn library.*

Formally, if we consider X as the set of independent variables (features) and y as the target, we can represent the data separation as shown in (Equations 3, 4):

(3) $$\left(X_{train},\;y_{train})=\;\{(X_{i},\;y_{i})|\;i\in Training\;set\right\}$$

(4) $$\left(X_{test},\;y_{test})=\;\{(X_{i},\;y_{i})|\;i\in Validation\;set\right\}$$

where $X_{train}$ and $y_{train}$ represent the features and targets of the training set, respectively, while $X_{test}$ and $y_{test}$ represent the features and targets of the validation set.

For each model, after training on the training set, we calculate evaluation metrics on the validation set to assess model performance. The evaluation function, denoted as Metric, measures the performance of the optimized model using the validation set observations, as shown in (Equation 5):

(5) $$Metric=\frac{1}{N}\;\overset{N}{\underset{i=1}{\sum }}L(f(X_{test,i\;},\;\theta_{opt}),\;y_{test,i})$$

Where $f(X_{test,i\;},\;\theta_{opt})$ is the model's prediction for test data point $X_{test,i\;}$, using the optimized parameters $\theta_{opt}$. $y_{test,i}$ represents the actual target value for $X_{test,i\;}$. *L* is a loss function that quantifies the difference between the prediction and the actual value (e.g., mean squared error for regression or cross-entropy for classification). *N* is the number of observations in the validation set.

Equations 3, 4 describe the division of data into training and validation sets, while (Equation 5) defines the evaluation metric to assess model performance after optimization. This approach ensures that the model is tested on unseen data, providing a reliable measure of its generalization capabilities.

### 3.3 Validation and optimization process for ML models

We explored a broad range of ML algorithms, including Gradient Boosting, Naive Bayes, Random Forest, AdaBoost, Logistic Regression, SVM, SGD, Decision Trees, and KNN, optimizing each to enhance the accuracy of predictions for mortality and hospitalization risks (31). Previous studies have demonstrated the effectiveness of ML in cardiovascular risk stratification, showing that these models outperform traditional methods in handling complex datasets and modeling non-linear relationships, thus providing higher sensitivity and specificity (32, 33). The implementation was carried out in a Python environment, using advanced libraries such as *pandas, numpy*, and *scikit-learn*, with a script that managed data loading, cleaning, and splitting for model training and validation.

The selected features include 10 numerical and 11 categorical variables, as outlined in Table 1. After dividing the dataset into a training set (70%) and a validation set (30%) using the *train_test_split* function from *scikit-learn*, we created pipelines for each model, applying feature standardization via *StandardScaler*. Feature standardization was performed using the following formula (Equation 6):

(6) $$X_{scaled}=\frac{X-\mu }{\sigma }\;$$

Where *X* represents the original value of the feature, μ is the mean of the feature values in the training set, σ is the standard deviation of the feature in the training set. This transformation scales the features to have a mean of zero and a standard deviation of one, improving the stability and performance of ML algorithms, especially those sensitive to data scaling.

We developed two distinct predictive models, focusing on mortality and hospitalization events as target variables for our patient cohort. Each model was trained separately on target-specific data and validated to ensure the reliability of the results. To minimize variance and improve the robustness of performance estimates, we applied 10-fold cross-validation, in line with established methods (34). The training process included a class balancing phase to address the data imbalance for mortality and hospitalization targets, a common issue in clinical datasets. Using SMOTE (Synthetic Minority Over-sampling Technique), we balanced the training set for each target by creating synthetic samples of the minority class, enhancing the models' ability to handle imbalanced data. This approach improved the sensitivity and specificity of the models, reducing the risk of misclassifying high-risk patients. The developed models were rigorously validated using standard metrics such as the AUC, accuracy, sensitivity, and specificity (35). For each model, we implemented a hyperparameter tuning phase using Python's *GridSearckCh,* a tool provided by the scikit-learn library that enables an exhaustive search for the optimal combination of hyperparameters to maximize model performance. *GridSearchCk* evaluates each combination specified in a predefined parameter grid, applying cross-validation to ensure that the performance obtained is representative and not overly dependent on the training data.

We used AUC as the primary metric for hyperparameter tuning, chosen because it represents the model's ability to correctly distinguish between classes, regardless of the classification threshold. AUC is particularly useful in medical contexts, where it is crucial to reduce both false positives and false negatives. A higher AUC indicates a more accurate model in predicting clinical events such as mortality and hospitalization, thereby improving the quality of therapeutic decision-making.

Formally, the optimization process aims to maximize AUC by selecting the optimal set of hyperparameters θ, and can be expressed as follows (Equation 7):

(7) $$\theta^{*}=arg\underset{\theta \in \Theta }{\max}AUC(f(X_{train};\;\theta ),\;y_{train})$$

Where θ∈Θrepresents the set of hyperparameter combinations specified in the search grid, $f(X_{train};\;\theta )$ is the model's predictive function trained on the training data $X_{train}$ with parameters θ, AUC is the evaluation metric that measures the area under the ROC curve, representing model performance relative to the true values $y_{train}$, $\theta^{*}$ is the combination of hyperparameters that maximizes AUC.

In Python, *GridSearchCV* applies cross-validation to each combination of hyperparameters θ, splitting the training set into *k* folds. The cross-validated mean AUC, denoted as AUCcv, for each fold can be expressed as (Equation 8):

(8) $$AUC_{cv}=\frac{1}{k}\overset{k}{\underset{i=1}{\sum }}AUC(\;f(X_{train_{i}};\;\theta ),\;y_{train_{i}})$$

Where $X_{train_{i}}$ and $y_{train_{i}}$ represent the training data and targets for the i-th fold, respectively, *k* is the number of folds in the cross-validation. At the end of the procedure, *GridSearchCV* returns the combination of hyperparameters $\theta^{*}$that maximizes the mean AUC across folds, providing an optimal configuration that represents the entire training set and minimizes the risk of overfitting. This approach ensures that the model is optimized for class discrimination, enhancing its generalizability to new data.

### 3.4 Selected ML models post-optimization

After the hyperparameter optimization process and using AUC as the primary metric to select the most effective model, Random Forest proved to be the best suited for predicting both the *Mortality* target (patient mortality event) and the *HF Hospitalization* target (patient hospitalization event). Model selection was based on comparing the average AUCs obtained through cross-validation for each model and target.

For predicting both the *Mortality* and *HF Hospitalization* targets, Random Forest showed optimal results. Random Forest is an ensemble learning method that builds multiple decision trees during training and combines their predictions to enhance the model's accuracy and robustness. The final prediction for each target using Random Forest, denoted as $f_{RF}(X)$, is obtained by averaging (for regression) or taking the majority vote (for classification) across the predictions from all trees, as shown in (Equation 9):

(9) $$f_{RF}(X)=\frac{1}{N}\overset{N}{\underset{\;j=1}{\sum }}f_{j}(X)$$

Where *N* is the number of decision trees in the forest, $f_{j}(X)$ represents the prediction of the j-th tree for input *X*.

Each tree is trained on a randomly sampled subset of the training data with replacement, optimizing specifically for the *Mortality* and *HF Hospitalization* targets. The aggregation of predictions enhances the model's generalization ability, reducing the risk of overfitting and stabilizing its capacity to accurately predict both mortality and hospitalization events.

### 3.5 Data measurements

In our study, predictive models effectively differentiate between survival and mortality outcomes among HF patients. These models categorize observations based on their predictions: an outcome is identified as either an accurate mortality prediction (TP—true positive), an accurate survival prediction (TN—true negative), an incorrectly predicted mortality (FP -false positive), or a missed mortality (FN—false negative). This classification is vital for assessing the model's accuracy and utility in clinical settings.

The model's performance is evaluated using several metrics, which are crucial for ensuring accurate and reliable predictions:

• *AUC-ROC (Area Under the Curve—Receiver Operating Characteristics)*: Measures the model's discriminative ability between outcome classes. The ROC curve plots the true positive rate (TPR) against the false positive rate (FPR) across varying thresholds u, and the AUC is calculated as (Equation 10):
  (10) $$AUC=\overset{1}{\underset{0}{\int }}TPR\;[FPR^{-1}(u)]\;du$$

This integral covers all possible decision thresholds, providing a comprehensive measure of predictive accuracy.

• *Accuracy*: Represents the ratio of correctly predicted instances (both true positives and true negatives) to the total number of instances evaluated. It is defined by the following (Equation 11):
  (11) $$Classification\;Accuracy=\;\frac{TP+TN}{TP+TN+FP+FN}$$
• *Precision*: Indicates the accuracy of positive predictions (mortality predictions), highlighting the model's ability to minimize false alarms, defined as (Equation 12):
  (12) $$Precision=\;\frac{TP}{TP+FP}$$
• *Recall (Sensitivity)*: Reflects the model's ability to identify all actual positive instances (actual mortalitys), which is crucial for ensuring that no high-risk patients are overlooked, defined as (Equation 13):
  (13) $$Recall=\;\frac{TP}{TP+FN}$$
• *F1 Score*: Combines precision and recall into a single metric, providing a balanced view of the model's overall predictive precision and sensitivity, defined as (Equation 14):
  (14) $$F1=\;2\;x\;\frac{Precision\;x\;Recall}{Precision+\;Recall}$$
• *Matthews Correlation Coefficient (MCC):* A comprehensive measure that takes into account true and false positives and negatives, offering a balanced metric even for imbalanced datasets. The MCC is especially valuable as it ranges from −1 (total disagreement between predictions and actuals) to +1 (perfect prediction), with 0 indicating no predictive power, defined as (Equation 15):
  (15) $$MCC=\;\frac{\left(TP\;x\;TN)-(FP\;x\;FN\right)}{\sqrt{\left(TP+\;FP)(TP+FN)(TN+\;FP)(TN+FN\right)}}$$

Utilizing these metrics ensures a thorough evaluation of the model's performance, facilitating improved clinical decision-making and patient management strategies in HF treatment. The integration of these diverse metrics, particularly AUC alongside precision, recall, and F1 score, supports the model's robustness, making it a valuable tool in clinical environments.

## 4 Results and discussion

In this section, we will discuss the selection of ML models used for risk prediction in patients with HF and thyroid dysfunctions, provide a detailed interpretation of the results for different thyroid subgroups, and introduce an experimental section on risk stratification. The objective is to explore the models' performance and evaluate their clinical applicability in the context of personalized risk management.

### 4.1 Performance of the selected predictive models

The results obtained from the optimized ML models for predicting mortality and hospitalization risks in patients with HF and thyroid dysfunctions are presented in Tables 2, 3. Each table includes a column labeled “Algorithm,” which lists the ML algorithms considered in this study. Various algorithms known for their effectiveness in classification tasks were selected, including Random Forest, Stochastic Gradient Descent (SGD), Logistic Regression, Support Vector Machines (SVM), Gradient Boosting, AdaBoost, Naive Bayes, Neural Network, K-Nearest Neighbors (KNN), and Decision Tree. This variety of algorithms allows for a comprehensive comparison of performance, both in terms of predictive accuracy and the ability to balance key metrics such as precision, recall, and F1-score.

**Table 2:** Model performance for mortality prediction**.**

Algorithm	AUC	Accuracy	F1	Precision	Recall	MCC
RandomForest	0.797	0.747	0.685	0.768	0.618	0.485
SGD	0.794	0.764	0.724	0.755	0.696	0.520
LogisticRegression	0.786	0.738	0.681	0.744	0.627	0.466
GradientBoosting	0.786	0.707	0.621	0.733	0.539	0.404
AdaBoost	0.762	0.721	0.660	0.721	0.608	0.430
SVM	0.759	0.729	0.667	0.738	0.608	0.448
NaiveBayes	0.753	0.690	0.585	0.725	0.490	0.369
NeuralNetwork	0.735	0.699	0.631	0.694	0.578	0.384
KNN	0.698	0.668	0.600	0.648	0.559	0.322
DecisionTree	0.608	0.624	0.522	0.603	0.461	0.227

**Table 3:** Model performance for HF hospitalization prediction**.**

Algorithm	AUC	Accuracy	F1	Precision	Recall	MCC
RandomForest	0.786	0.703	0.638	0.652	0.625	0.387
NeuralNetwork	0.785	0.725	0.659	0.685	0.635	0.430
LogisticRegression	0.784	0.729	0.687	0.667	0.708	0.449
SVM	0.779	0.725	0.683	0.660	0.708	0.442
NaiveBayes	0.769	0.690	0.643	0.621	0.667	0.370
SGD	0.763	0.712	0.673	0.642	0.708	0.418
GradientBoosting	0.746	0.681	0.597	0.635	0.563	0.336
KNN	0.727	0.664	0.645	0.579	0.729	0.342
AdaBoost	0.721	0.690	0.632	0.629	0.635	0.364
DecisionTree	0.641	0.659	0.606	0.588	0.625	0.307

The performance of each algorithm was evaluated using metrics such as the AUC, accuracy, F1-score, precision, recall, and Matthews Correlation Coefficient (MCC). The AUC metric was particularly emphasized as the primary indicator of model performance, guiding the interpretation of results.

For mortality prediction, the Random Forest model achieved the best performance with an AUC of 0.797, an accuracy of 74.7%, and an F1-score of 0.685. These values indicate a good ability of the model to discriminate between high-risk and low-risk patients, balancing precision (0.768) and recall (0.618). The MCC for Random Forest was 0.485, further supporting its balanced performance across classes. This combination suggests that Random Forest is effective in identifying at-risk patients while maintaining a low rate of false positives, making it particularly suitable for mortality prediction.

For hospitalization risk prediction, the Random Forest model again demonstrated the best performance, with an AUC of 0.786, an accuracy of 70.3%, and an F1-score of 0.638. With a precision of 0.652, recall of 0.625, and an MCC of 0.387, Random Forest effectively identifies patients at risk of hospitalization, maintaining a favorable balance between accuracy and sensitivity. This model's reliability for predicting hospitalization risk makes it a valuable tool for clinical applications where capturing at-risk patients is essential, even if it involves a slightly higher rate of false positives.

In summary, the results in Tables 2, 3 indicate that the Random Forest model is particularly promising for predicting both mortality and hospitalization risks. The AUC metric, used as the primary indicator, confirms the effectiveness of this model in providing robust decision support in clinical settings. Its application could significantly improve risk stratification and personalize treatments for patients with HF and thyroid dysfunctions, contributing to more precise and patient-centered medicine.

**Figure 2:**
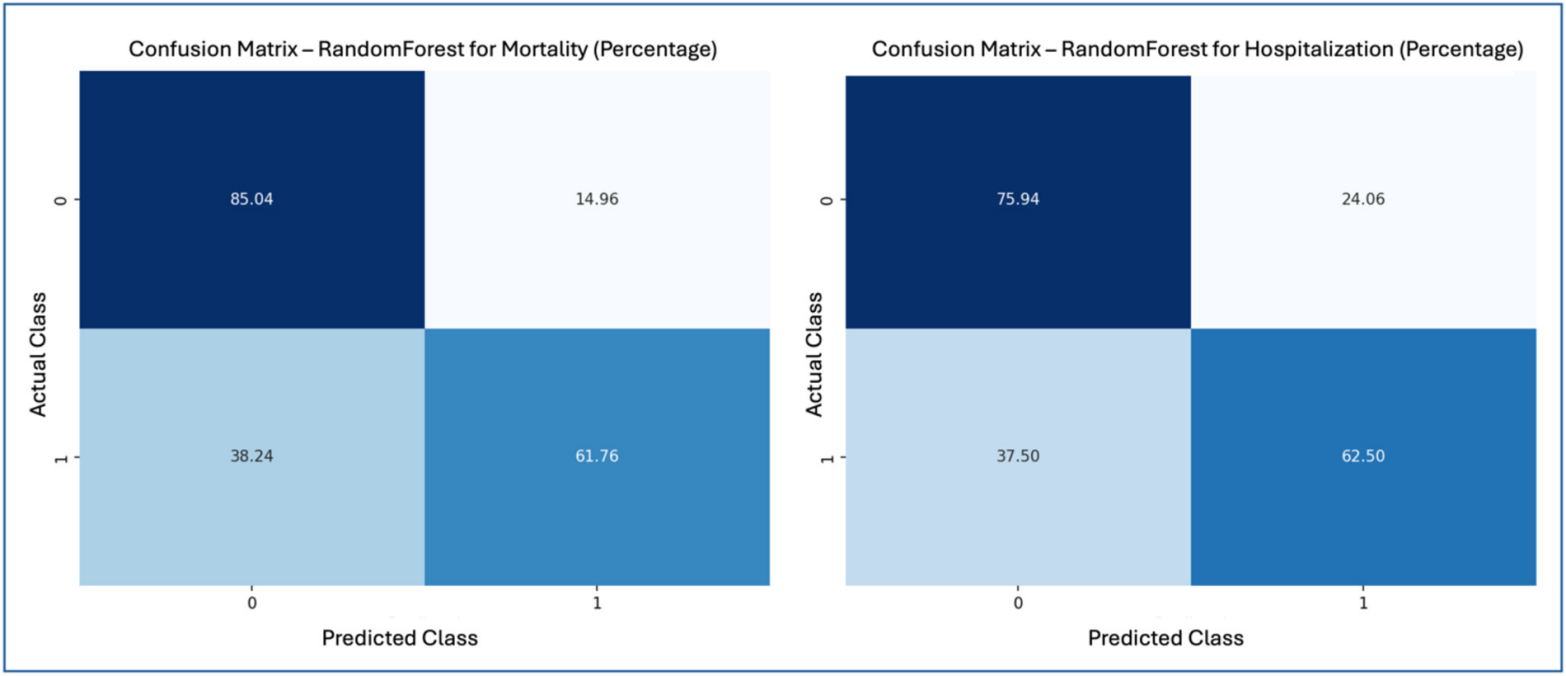
Confusion matrices—random forest for mortality and hospitalization.

Figure 2 presents the confusion matrices for the top-performing ML model in predicting mortality and hospitalization risks, both achieved using the Random Forest algorithm: mortality prediction (left) and hospitalization prediction (right). These matrices are displayed in percentages, offering a comprehensive view of model performance regarding correct classifications and error rates. In the mortality prediction matrix (left), the Random Forest model correctly identified 85.04% of low-risk patients (class 0), while 14.96% of these patients were incorrectly classified as high-risk. For the high-risk group (class 1), the model correctly classified 61.76% of patients but misclassified 38.24% as low-risk. These results indicate that, while the Random Forest model has high precision for predicting low-risk patients, its sensitivity in identifying high-risk cases is moderate.

For hospitalization prediction (right), the Random Forest model accurately classified 75.94% of patients not at risk (class 0), with 24.06% misclassified as at-risk. In the at-risk group (class 1), 62.50% of patients were correctly identified, while 37.50% were classified as false negatives. This performance shows that the Random Forest model is effective in predicting hospitalization risk, maintaining a reasonable balance between precision and recall for at-risk patients.

Figure 2 illustrates the strengths and limitations of the Random Forest model in both predictive tasks. The model shows high accuracy for the low-risk mortality class but misses a significant portion of high-risk cases. Similarly, it performs well in predicting hospitalization risk but also exhibits some false negatives within the high-risk group. The model demonstrates a satisfactory balance between accuracy and sensitivity, reinforcing its clinical applicability for risk stratification.

**Figure 3:**
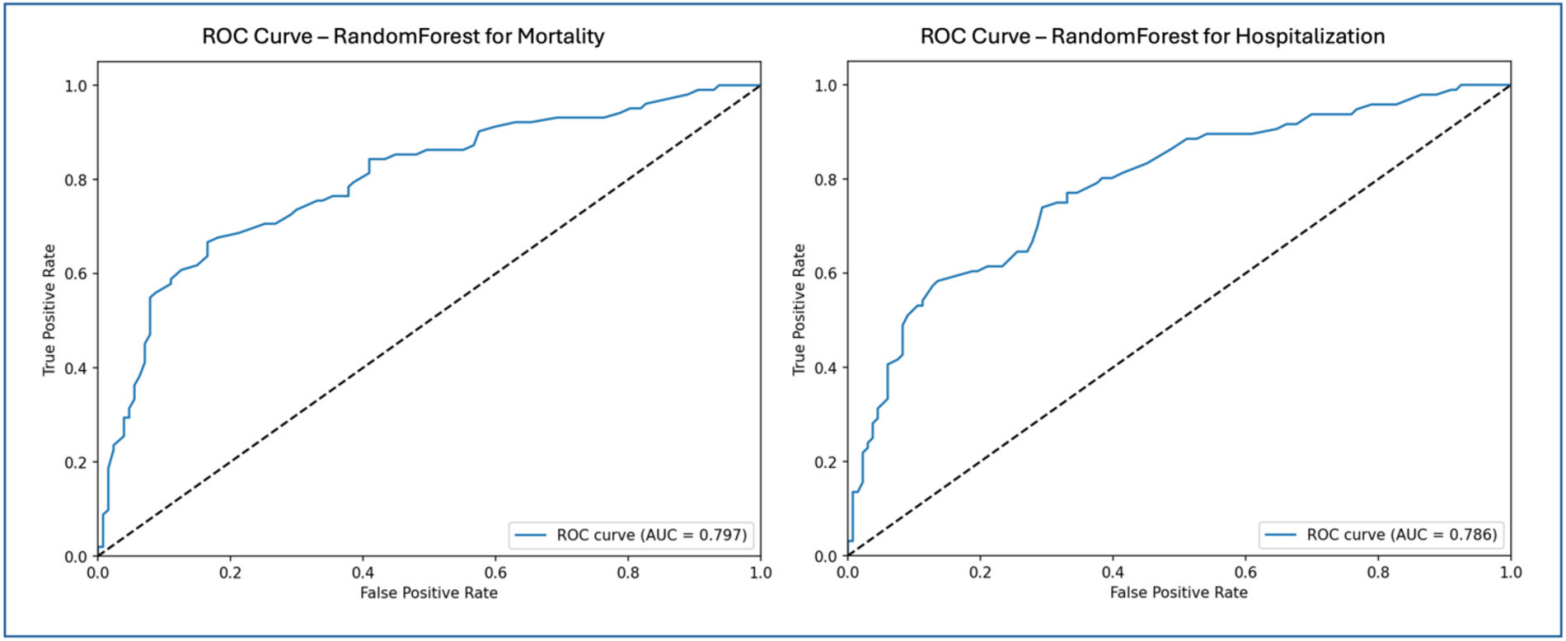
ROC curves—random forest for mortality and hospitalization.

Figure 3 shows the Receiver Operating Characteristic (ROC) curves for the Random Forest model in predicting mortality and hospitalization risks: mortality prediction (left) and hospitalization prediction (right). The ROC curve illustrates the model's ability to distinguish between classes, plotting the relationship between True Positive Rate (Sensitivity) and False Positive Rate. The Area Under the Curve reflects model performance, where values closer to 1 indicate greater discriminatory power. For mortality prediction, the Random Forest model achieved an AUC of 0.797, as depicted in the left ROC curve, demonstrating a strong capability to differentiate between high and low mortality risk. The ROC curve remains well above the reference line (indicating random classification) across thresholds, showcasing the Random Forest model's ability to sustain a high True Positive Rate while minimizing False Positives. For hospitalization prediction, the Random Forest model achieved an AUC of 0.786, as shown in the right ROC curve. Although slightly lower than the AUC for mortality prediction, this value still reflects strong performance in identifying hospitalization risk. The ROC curve for the Random Forest model stays above the reference line, indicating good model sensitivity and specificity in distinguishing hospitalized from non-hospitalized patients.

Figure 3 highlights the effective performance of the Random Forest model in both prediction tasks. The AUC values for mortality and hospitalization predictions confirm the model's suitability for clinical risk stratification. The ROC curves emphasize the model's capacity to balance True Positive and False Positive rates, reinforcing its utility as a reliable tool for clinical decision-making in managing patients with HF and thyroid dysfunction.

### 4.2 Analysis of clinical and statistical differences among thyroid subgroups

Among the 762 patients analyzed, 187 were affected by hypothyroidism; of these, 93 had a prior history of hypothyroidism, while in 94 cases, hypothyroidism was diagnosed during the initial or subsequent evaluations at our center. LT3 syndrome was diagnosed in 15 patients, while a total of 58 patients had hyperthyroidism, with 46 having a prior history and 12 diagnosed at the time of the first evaluation or during follow-up.

**Figure 4:**
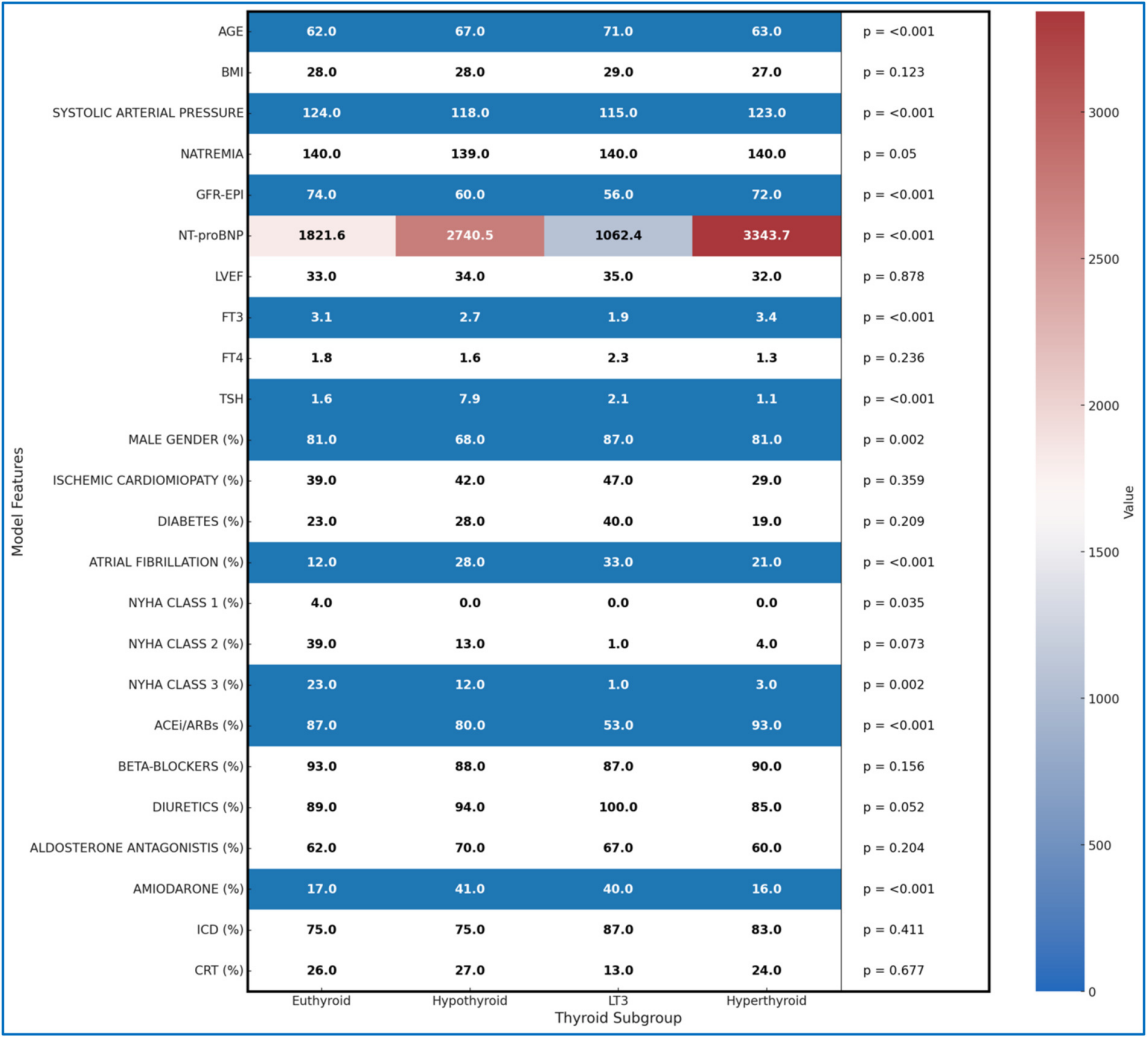
Heatmap of all clinical features by thyroid subgroup.

Figure 4 presents the statistical characteristics of the patients, divided into subgroups based on the presence or absence of thyroid disorders, providing a detailed overview of demographic variables, risk factors, and ongoing therapies for each subgroup. This arrangement allows for an in-depth comparison of clinical differences among patients with various thyroid dysfunctions. Among the patients, 175 were on amiodarone therapy at the time of the initial evaluation: 63 for secondary prevention of supraventricular tachycardia or flutter/atrial fibrillation, 73 for secondary prevention of sustained ventricular tachycardia/ventricular fibrillation, 24 for both, and 15 for control of frequent supraventricular or ventricular ectopic beats. To compare characteristics across the different thyroid groups, the Kruskal–Wallis test was used, a non-parametric test suitable for variables that do not follow a normal distribution. This statistical method allows significant differences to be detected among multiple groups without assuming normality, which is particularly useful given the nature of clinical variables, which are both continuous and categorical. In the heatmap (Figure 4), significant differences (*p* < 0.005) are visually highlighted using a blue background with white text, allowing immediate identification of key variables. Additionally, NT-proBNP values are color-coded using a gradient that reflects their magnitude in relation to the scale shown in the accompanying color bar, facilitating intuitive comparison across subgroups.

The results indicate that the mean age differs significantly between groups (*p* < 0.001), with patients with LT3 syndrome being older on average (71 years) than euthyroid patients (62 years). Systolic blood pressure and renal function, measured by GFR-EPI, also show significant differences (*p* < 0.001); hypothyroid and LT3 patients have lower average values, suggesting possible involvement of cardiovascular and renal function. NT-proBNP levels, an indicator of HF severity, are significantly higher in hypothyroid and hyperthyroid patients compared to euthyroid patients, reflecting a higher degree of clinical impairment (*p* < 0.001).

Thyroid function parameters, such as FT3 and TSH, also differ significantly among the groups. LT3 patients have the lowest average FT3 levels compared to the other subgroups, while hypothyroid patients show elevated TSH levels (*p* < 0.001). Atrial fibrillation is more common in patients with thyroid dysfunctions, particularly among those with LT3 and hypothyroidism, with percentages of 33% and 28%, respectively, compared to euthyroid patients (12%), suggesting an increased predisposition to arrhythmic events in the presence of thyroid disorders (*p* < 0.001).

The distribution of patients across NYHA classes reveals further differences, with lower representation of thyroid dysfunction patients in the more advanced classes (*p* = 0.002), potentially reflecting a different severity of symptoms among groups. In terms of pharmacological therapies, hypothyroid and LT3 patients are more frequently treated with diuretics and amiodarone compared to euthyroid patients, with statistically significant differences for the use of ACEi/ARBs and amiodarone (*p* < 0.001), which may indicate specific therapeutic needs for these subgroups.

These differences between thyroid groups provide a deeper understanding of the distinctive clinical profiles associated with thyroid dysfunctions, highlighting how clinical risk and therapeutic needs may vary based on thyroid status. The detailed statistical breakdown in Figure 4, along with the Kruskal–Wallis test, provides valuable information for a better understanding of the clinical specificities of each group, supporting the implementation of more targeted therapeutic strategies.

### 4.3 Interpretation of model results with LIME for thyroid subgroups

**Figure 5:**
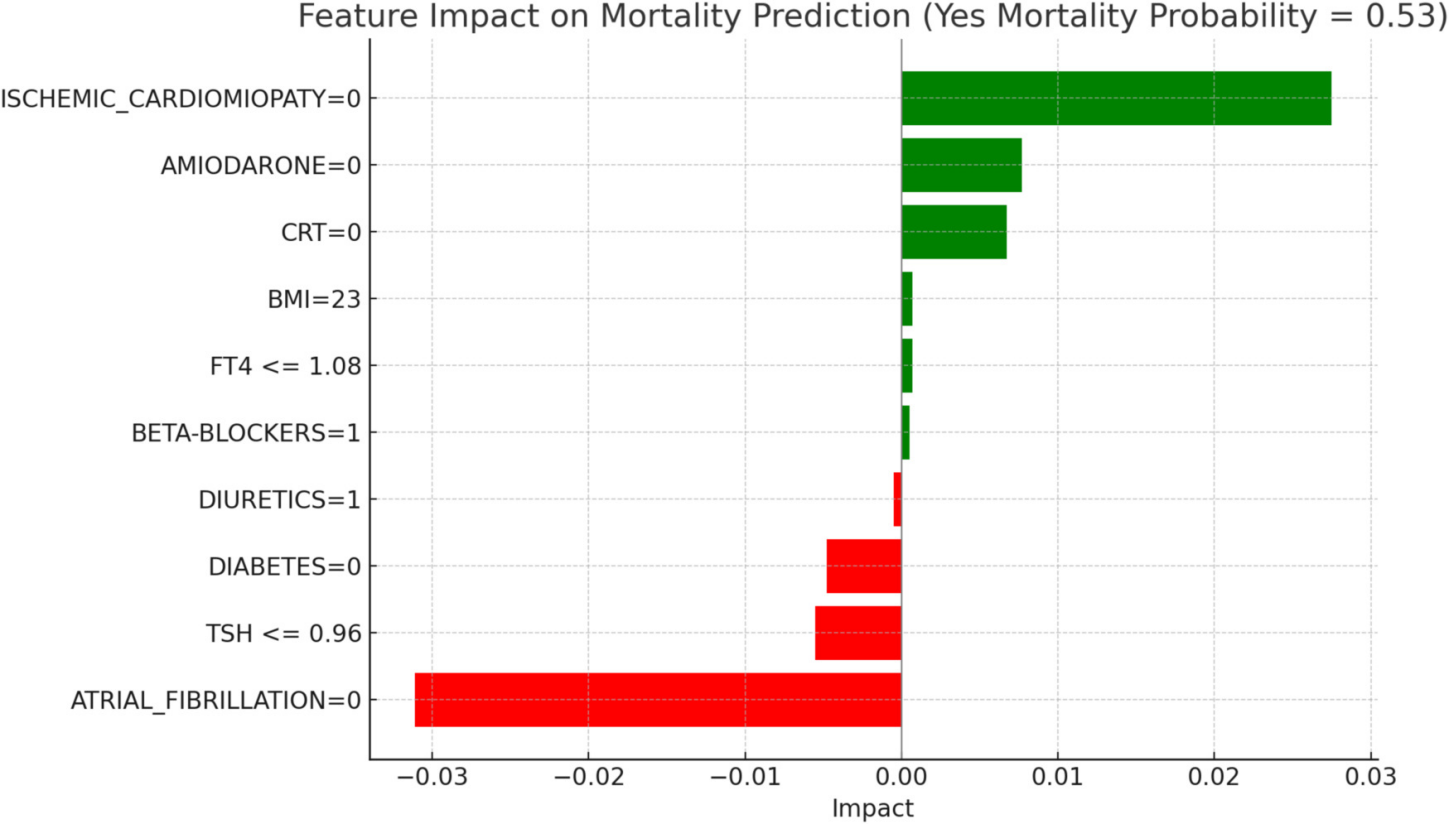
LIME explanations for mortality prediction model in euthyroid patients.

**Figure 6:**
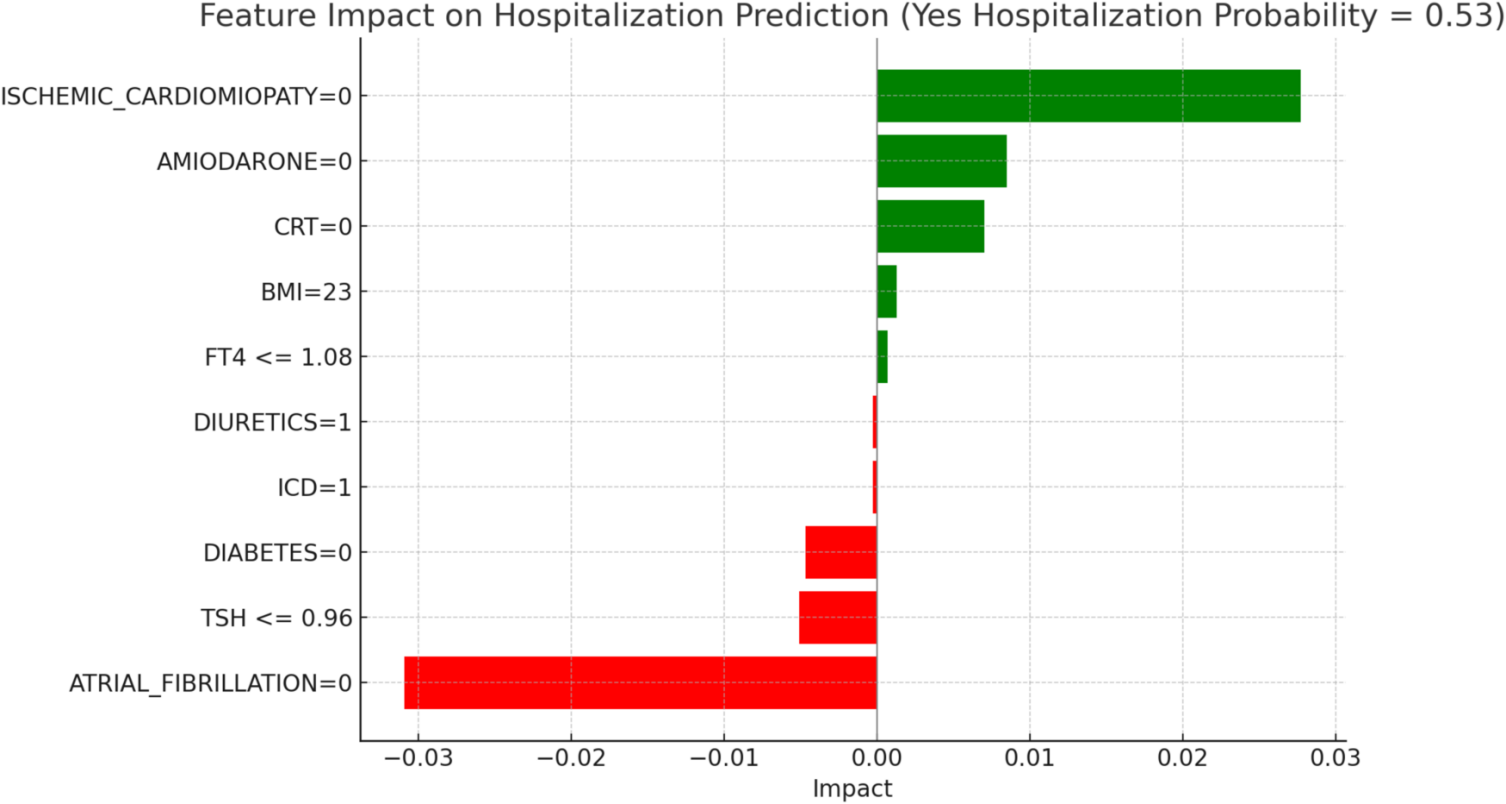
LIME explanations for hospitalization prediction model in euthyroid patients.

**Figure 7:**
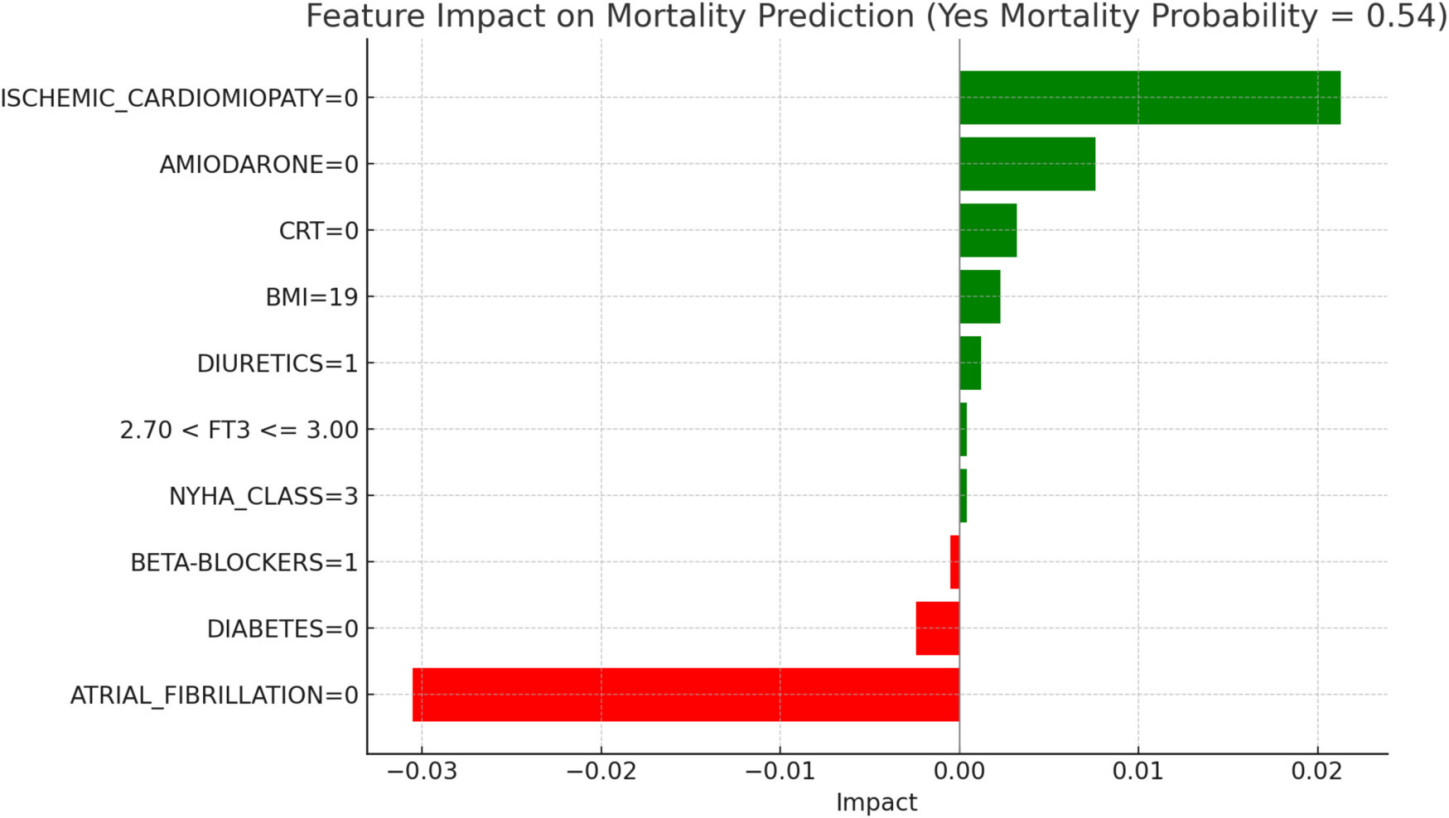
LIME explanations for mortality prediction model in hypothyroid patients.

**Figure 8:**
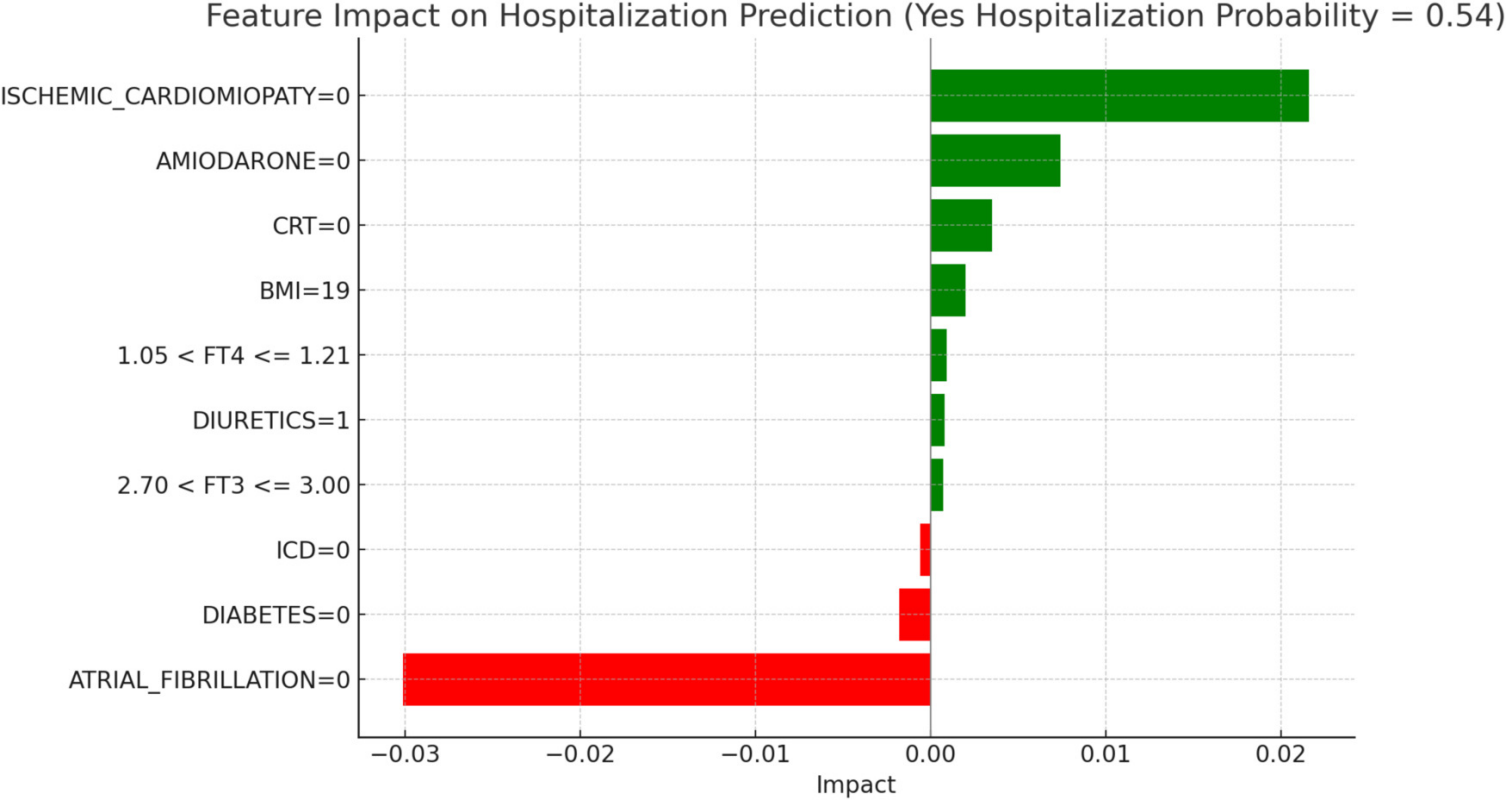
LIME explanations for hospitalization prediction model in hypothyroid patients.

**Figure 9:**
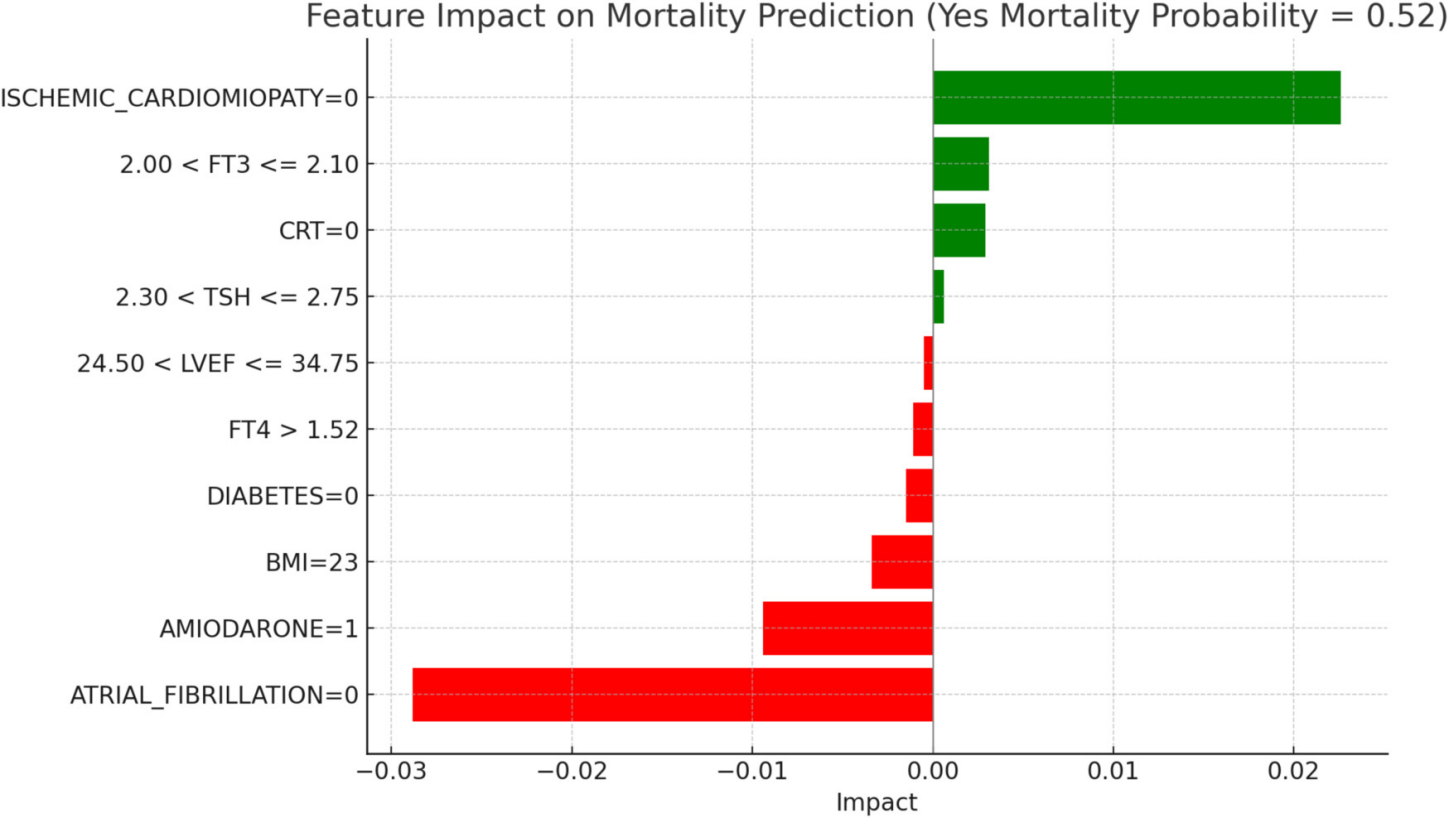
LIME explanations for mortality prediction model in LT3 patients.

**Figure 10:**
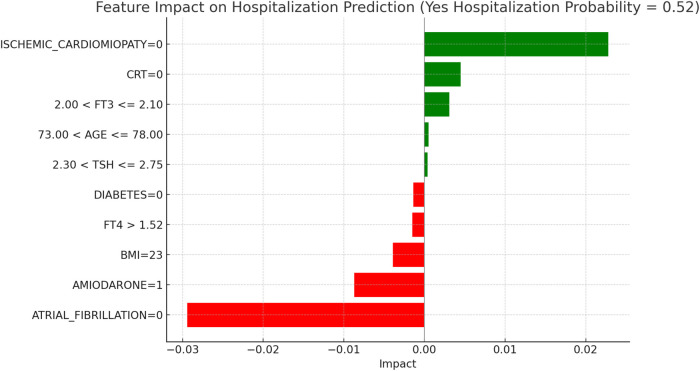
LIME explanations for hospitalization prediction model in LT3 patients.

**Figure 11:**
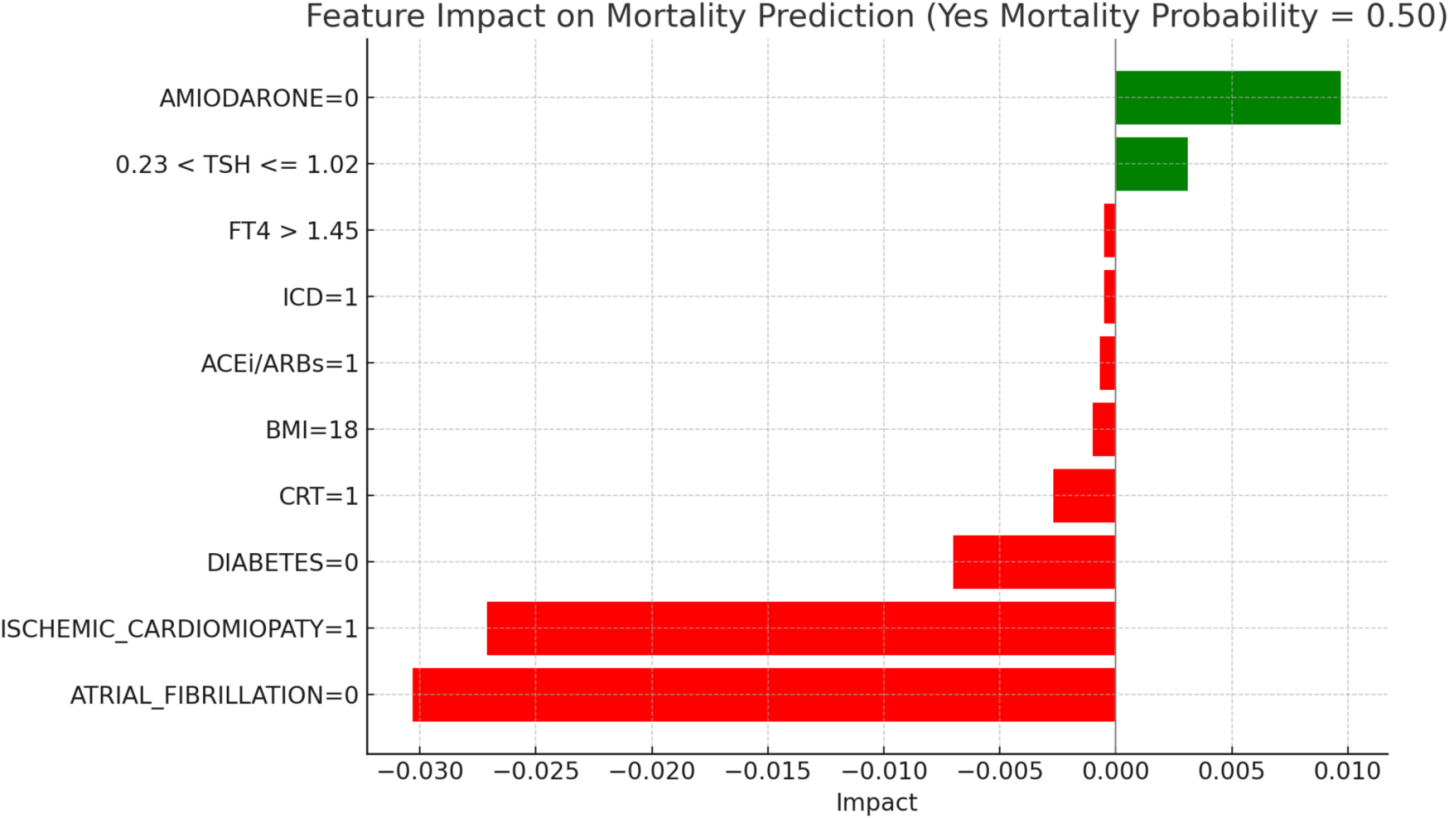
LIME explanations for mortality prediction model in hyperthyroid patients.

**Figure 12:**
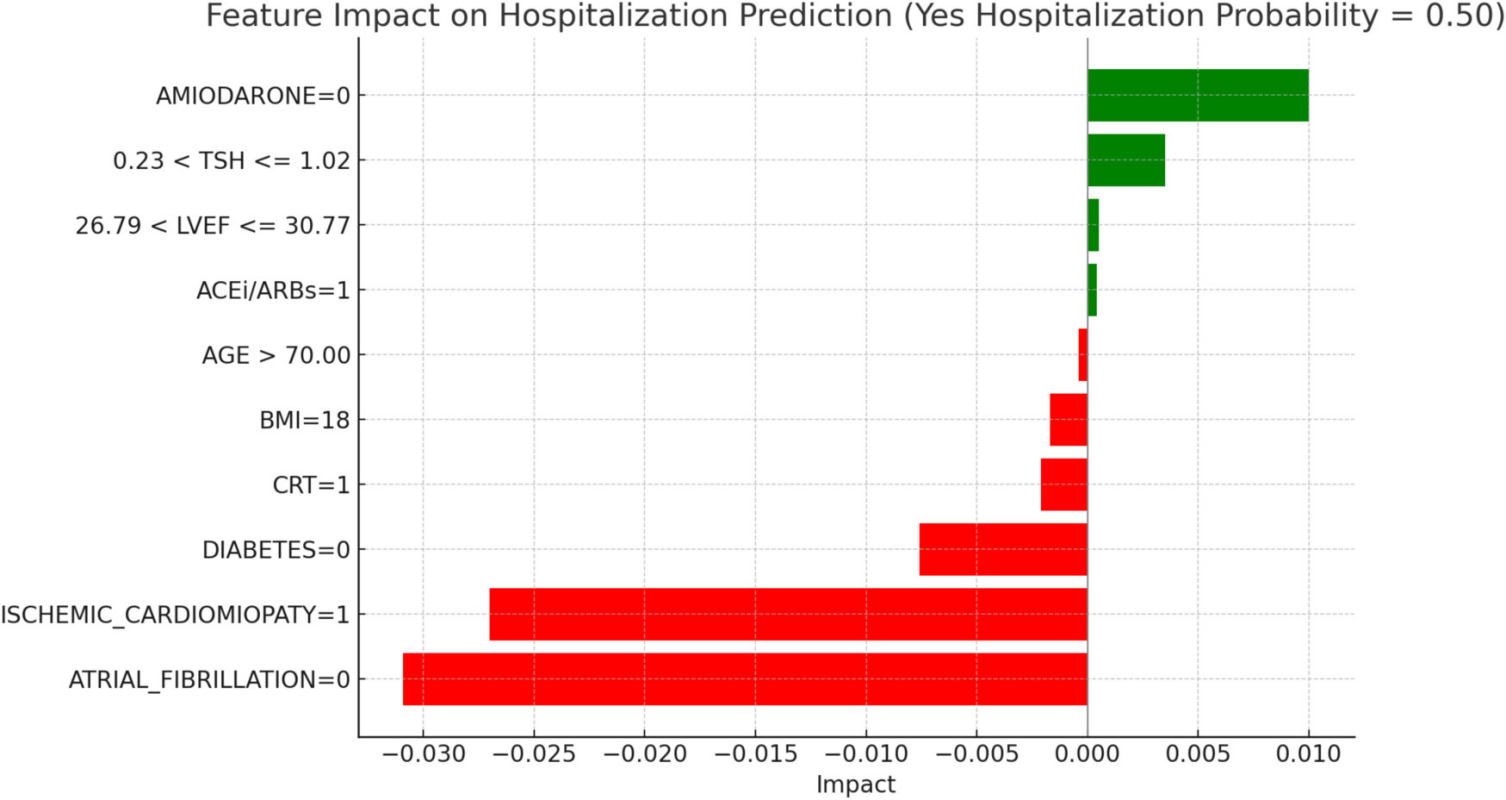
LIME explanations for hospitalization prediction model in hyperthyroid patients.

This section applies the Local Interpretable LIME technique to interpret the Random Forest model results, focusing on specific subgroups within thyroid-related patient populations. LIME enables the interpretation of complex models by creating locally interpretable models around individual predictions, allowing us to examine the contribution of each variable to the model's final decisions. The LIME technique was applied uniformly across all thyroid-related subgroups to support the interpretability of the model predictions. For each subgroup, the approach enabled the identification of clinical variables such as atrial fibrillation, ischemic cardiomyopathy, pharmacological treatment, and thyroid hormone values, contributing to the estimated risks of mortality and hospitalization. Illustrative examples of these explanations are presented in Figures 4, 5–11, including euthyroid, hypothyroid, LT3 and Hyperthyroid patient groups, thus offering a consistent interpretation framework across the cohort. In the graphical representations (Figures 5–11, 12), the impact of each clinical feature is visually represented through color-coded horizontal bars. Specifically, green bars indicate features that contribute to an increase in the predicted probability of the outcome (e.g., mortality or hospitalization), suggesting a higher risk associated with those variables. Conversely, red bars represent features that reduce the predicted probability, thus being protective factors associated with a lower risk. This visual distinction enhances interpretability by allowing a quick understanding of whether each feature pushes the model prediction toward or away from a critical outcome.

**Figure 13:**
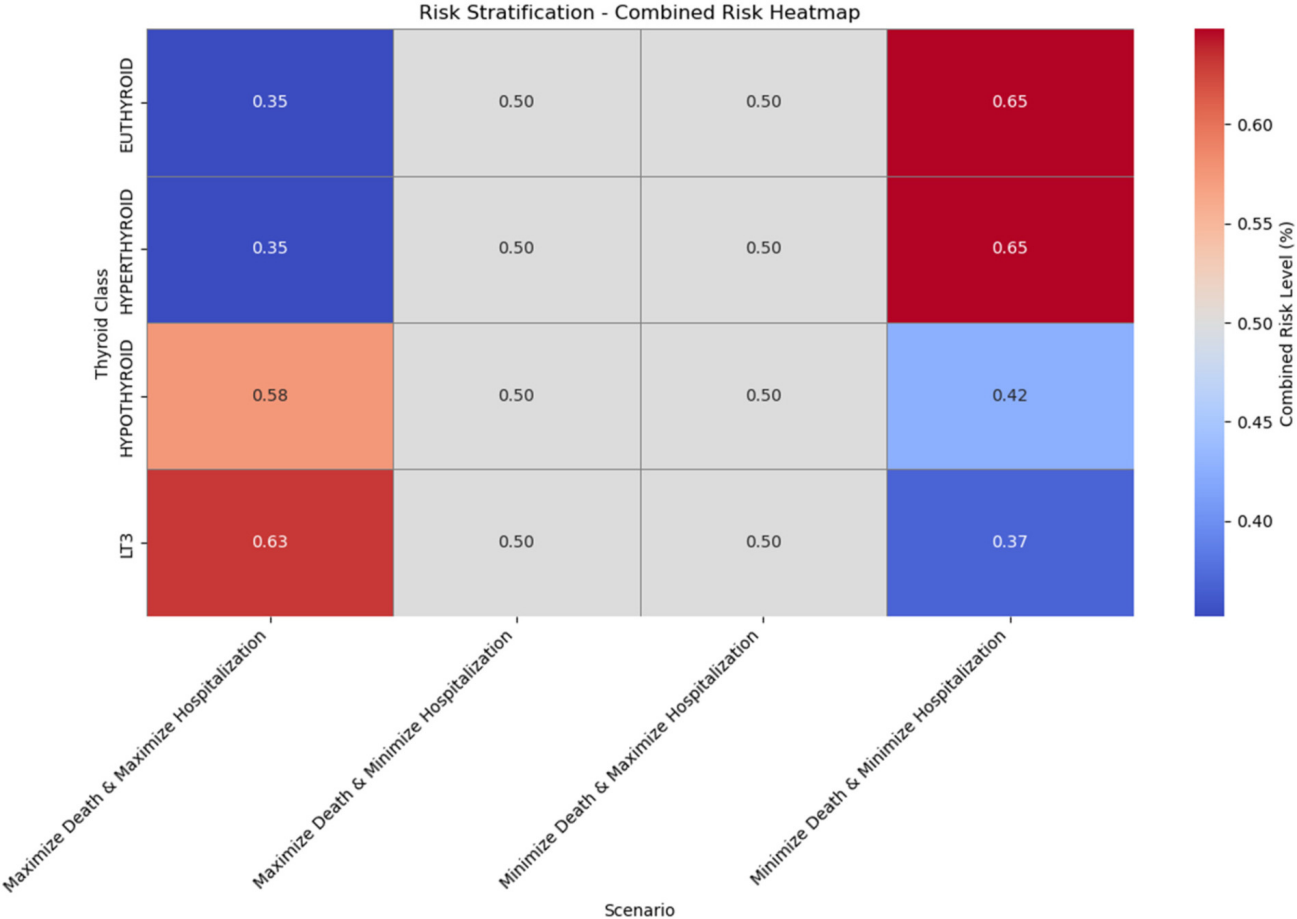
Risk stratification—combined risk heatmap across thyroid classes and scenarios.

For each thyroid subgroup, LIME was applied to generate explanations that illustrate how key clinical factors modulate the model's predictions vary based on key clinical features, such as TSH levels, T3 and T4 hormone concentrations, and patient demographics. By analyzing these explanations, we can gain a clearer understanding of which features drive the model's predictions for each thyroid subgroup, distinguishing between low and high-risk classifications for both mortality and hospitalization.

Figures 5, 6 present the LIME interpretation results for the mortality and hospitalization models, respectively, in euthyroid patients. These figures list the main clinical features that impact the model's predictions. The impact values reflect the influence of each feature on the predicted probability, with positive values indicating features that contribute toward the outcome (e.g., mortality or hospitalization), while negative values indicate protective associations.

In Figure 5, titled “LIME Explanations for Mortality Prediction Model in Euthyroid Patients,” the model shows a 53%predicted probability for “YES MORTALITY” vs. 47% for “NO MORTALITY,” suggesting a slight inclination toward mortality for this subgroup. Among the influential features, the absence of atrial fibrillation (ATRIAL_FIBRILLATION = 0) shows a protective effect with an impact of −0.0311, lowering the mortality probability. Conversely, the absence of ischemic cardiomyopathy (ISCHEMIC_CARDIOMYOPATHY = 0) slightly increases the likelihood of mortality, with an impact value of 0.0275. Other features contribute with varying, though smaller, effects. For instance, the absence of the medication Amiodarone (AMIODARONE = 0) and of cardiac resynchronization therapy (CRT = 0) display minor positive impacts of 0.0077 and 0.0067, respectively, indicating an association with increased mortality when these treatments are not administered. Lower levels of TSH (≤0.96) reduce the probability of mortality with an impact of −0.0055, while the absence of diabetes (DIABETES = 0) has a similarly protective effect, with an impact of −0.0048. Minimal impacts are observed for free T4 levels (FT4 ≤1.08), BMI (23), and the use of diuretics and beta-blockers, with values ranging between 0.0005 and 0.0007, suggesting a more subtle influence on mortality risk in this model.

Figure 6, “LIME Explanations for Hospitalization Prediction Model in Euthyroid Patients,” presents results for hospitalization prediction with identical predicted probabilities to the mortality model (53% for “YES HOSPITALIZATION” and 47% for “NO HOSPITALIZATION”), indicating a similar risk profile in this patient subgroup.

The absence of atrial fibrillation (ATRIAL_FIBRILLATION = 0) has a protective impact, reducing the likelihood of hospitalization with an impact value of −0.0309. Conversely, the absence of ischemic cardiomyopathy (ISCHEMIC_CARDIOMYOPATHY = 0) slightly increases the risk, showing a positive impact of 0.0277. The absence of Amiodarone (AMIODARONE = 0) and CRT (CRT = 0) also contribute to an increased hospitalization probability, with impact values of 0.0085 and 0.0070, respectively. Lower TSH levels (≤0.96) provide a protective influence with an impact of −0.0051, while the absence of diabetes (DIABETES = 0) similarly reduces the likelihood of hospitalization, reflected by an impact of −0.0047. BMI of 23 has a minor positive influence of 0.0013, indicating a slightly increased hospitalization probability for patients with this BMI value. Additional features with minimal impacts include free T4 levels (FT4 ≤1.08), presence of an ICD (ICD = 1), and the use of diuretics (DIURETICS = 1), each with values of 0.0007, −0.0003, and −0.0003 respectively. These factors suggest a nuanced, though limited, influence on the overall hospitalization prediction compared to the primary variables in this model.

Figure 7, “LIME Explanations for Mortality Prediction Model in Hypothyroid Patients,” presents the model's interpretation results for the mortality prediction in hypothyroid patients, with 54% predicted probability for “YES MORTALITY” and 46% for “NO MORTALITY,” indicating a slight inclination toward mortality in this group.

In this model, the absence of atrial fibrillation (ATRIAL_FIBRILLATION = 0) serves as a protective factor, reducing the mortality probability with an impact of −0.0305. On the other hand, the absence of ischemic cardiomyopathy (ISCHEMIC_CARDIOMYOPATHY = 0) slightly increases the mortality risk, with a positive impact of 0.0213. The lack of Amiodarone (AMIODARONE = 0) and CRT (CRT = 0) also contribute to an elevated mortality probability, with impacts of 0.0076 and 0.0032, respectively. Other clinical variables influence mortality predictions to a lesser degree. The absence of diabetes (DIABETES = 0) decreases mortality risk, with an impact of −0.0024, while a BMI of 19 has a slight positive effect of 0.0023, indicating a marginal association with increased mortality. The use of diuretics (DIURETICS = 1) and beta-blockers (BETA-BLOCKERS = 1) exert small impacts, with values of 0.0012 and −0.0005, respectively, highlighting their limited role in influencing mortality predictions. Additional factors, such as NYHA class (NYHA_CLASS = 3) and FT3 levels within the range 2.70 < FT3 ≤ 3.00, have minimal impacts of 0.0004 each, suggesting a nuanced but relatively insignificant influence on the model's overall prediction for mortality. In hypothyroid patients, the predicted probability of mortality was 54 percent. The absence of atrial fibrillation emerged as the most protective factor, aligning with its recognized clinical relevance in heart failure prognosis. Conversely, the absence of ischemic cardiomyopathy contributed to a moderate increase in predicted mortality, potentially reflecting the influence of alternative etiologies. Other variables, such as the lack of amiodarone therapy, absence of CRT, and a low BMI value, were associated with slightly elevated risk. FT3 values within borderline ranges and NYHA class exerted minor effects, confirming the multifactorial nature of mortality risk in this subgroup.

Figure 8, “LIME Explanations for Hospitalization Prediction Model in Hypothyroid Patients,” outlines the hospitalization prediction for hypothyroid patients, with 54% probability for “YES HOSPITALIZATION” and 46% for “NO HOSPITALIZATION,” again indicating a slight model tendency towards predicting hospitalization.

Key protective factors include the absence of atrial fibrillation (ATRIAL_FIBRILLATION = 0), which reduces the hospitalization risk with an impact of −0.0301. Meanwhile, the absence of ischemic cardiomyopathy (ISCHEMIC_CARDIOMYOPATHY = 0) slightly increases the hospitalization likelihood, with an impact of 0.0216. The absence of Amiodarone (AMIODARONE = 0) and CRT (CRT = 0) contribute positively, with impacts of 0.0074 and 0.0035, respectively, indicating that their absence may slightly increase hospitalization risk. Further influencing factors include BMI of 19, which has a minor positive impact of 0.0020 on hospitalization probability, and the absence of diabetes (DIABETES = 0), which has a small protective effect with an impact of −0.0018. Free T4 levels within the range 1.05 < FT4 ≤ 1.21 and FT3 levels within 2.70 < FT3 ≤ 3.00 add slight positive contributions, with impacts of 0.0009 and 0.0007, respectively. Finally, the presence of an ICD (ICD = 0) serves as a minor protective factor, with an impact of −0.0006, while diuretic usage (DIURETICS = 1) has a modest positive effect of 0.0008. These features, though present, exert relatively small effects in comparison to the more influential clinical factors impacting hospitalization predictions in this subgroup. In hypothyroid patients, the LIME interpretation results suggest a moderate increase in hospitalization risk, with a predicted probability of 54%. The absence of atrial fibrillation emerged as the most protective factor, consistent with its known adverse prognostic role in heart failure populations. Conversely, the absence of ischemic cardiomyopathy contributed positively to the predicted probability, potentially indicating the clinical impact of non-ischemic HF phenotypes in this subgroup. The absence of amiodarone and CRT therapy also showed modest positive contributions, aligning with the established utility of these interventions in selected HF patients. A lower BMI (19) was associated with a slight increase in predicted hospitalization, in line with the “obesity paradox” described in HF literature. Additionally, borderline FT4 and FT3 values exerted limited but noticeable effects, confirming the relevance of thyroid hormone levels in influencing short-term outcomes in this subgroup.

Figure 9, “LIME Explanations for Mortality Prediction Model in LT3 Patients,” shows the model's interpretation results for mortality prediction in LT3 patients, with a predicted probability of 52% for “YES MORTALITY” and 48% for “NO MORTALITY,” indicating a slight inclination towards predicting mortality for this group. Among the significant features, the absence of atrial fibrillation (ATRIAL_FIBRILLATION = 0) reduces the likelihood of mortality, acting as a protective factor with an impact of −0.0288. Conversely, the absence of ischemic cardiomyopathy (ISCHEMIC_CARDIOMYOPATHY = 0) slightly increases the probability of mortality, with a positive impact of 0.0226. Additionally, the use of Amiodarone (AMIODARONE = 1) appears to lower the mortality risk, indicated by an impact of −0.0094. BMI at 23 also has a slight protective influence, with an impact of −0.0034, while free T3 (FT3) levels in the range 2.00 < FT3 ≤ 2.10 contribute positively to mortality risk, showing an impact of 0.0031. The absence of CRT (CRT = 0) adds a minor positive influence with an impact of 0.0029, suggesting a potential association with increased mortality in LT3 patients when CRT is not in place. Other features play smaller roles: the absence of diabetes (DIABETES = 0) has a slight protective effect on mortality with an impact of −0.0015, and high levels of FT4 (>1.52) further reduce the probability of mortality with an impact of −0.0011. Additional factors, such as TSH levels between 2.30 and 2.75 and LVEF (Left Ventricular Ejection Fraction) values within 24.50–34.75, contribute minimally to the model's mortality predictions, with impacts of 0.0006 and −0.0005 respectively. Among LT3 patients, the model indicated a 52% probability of mortality. The strongest protective effect was associated with the absence of atrial fibrillation, while the absence of ischemic cardiomyopathy slightly increased predicted risk. The presence of amiodarone was linked to a lower mortality probability, possibly reflecting its therapeutic role in rhythm control. Hormonal indicators such as FT3 in the range 2.00–2.10 and higher FT4 levels provided subtle but consistent contributions. Overall, the results illustrate the complex interplay between metabolic, structural, and treatment-related factors in shaping risk within this distinct population.

Figure 10, “LIME Explanations for Hospitalization Prediction Model in LT3 Patients,” provides insights into the model's predictions for hospitalization within this group. The model shows a 52% predicted probability for “YES HOSPITALIZATION” and 48% for “NO HOSPITALIZATION,” again reflecting a slight tendency towards hospitalization risk. The absence of atrial fibrillation (ATRIAL_FIBRILLATION = 0) has the strongest protective effect, reducing the probability of hospitalization with an impact of −0.0294. In contrast, the absence of ischemic cardiomyopathy (ISCHEMIC_CARDIOMYOPATHY = 0) is associated with a slight increase in hospitalization likelihood, with an impact of 0.0228. Use of Amiodarone (AMIODARONE = 1) similarly lowers the hospitalization risk, shown by an impact of −0.0087. The absence of CRT (CRT = 0) shows a positive influence on hospitalization probability with an impact of 0.0045, while BMI at 23 has a protective impact with a value of −0.0039. Free T3 levels within 2.00 < FT3 ≤ 2.10 contribute a minor positive influence on hospitalization, with an impact of 0.0031, indicating a small association with increased risk for patients in this range. Other variables include FT4 levels greater than 1.52, which lower hospitalization probability with an impact of −0.0015, and the absence of diabetes (DIABETES = 0), which also acts protectively with an impact of −0.0014. Age within 73.00–78.00 years and TSH levels in the range 2.30 < TSH ≤ 2.75 exert minimal positive influences on hospitalization, with impacts of 0.0005 and 0.0004, respectively, suggesting limited yet present contributions in the model's hospitalization prediction. In LT3 syndrome patients, the predicted probability of hospitalization was 52%, indicating a subtle shift towards higher risk in this group. The absence of atrial fibrillation was again the most significant protective variable. Notably, the presence of amiodarone was associated with a lower predicted risk, which may reflect its therapeutic role in arrhythmia management among patients with compromised metabolic status. The absence of CRT demonstrated a minor positive impact on hospitalization probability, in line with its potential benefits in patients with advanced HF and electrical dyssynchrony. BMI at 23 appeared to exert a small protective influence, while FT3 values in the 2.00–2.10 range were associated with a mild increase in risk, consistent with reduced metabolic activity typical of LT3. Other features, including elevated FT4, absence of diabetes, and mid-range TSH values, showed marginal impacts, reinforcing the multifactorial nature of hospitalization risk in this complex subgroup.

Figure 11, “LIME Explanations for Mortality Prediction Model in Hyperthyroid Patients,” shows the model's interpretation results for mortality prediction in hyperthyroid patients, with a predicted probability split evenly at 50% for “YES MORTALITY” and 50% for “NO MORTALITY,” indicating no strong inclination towards either outcome in this group.

Key protective factors include the absence of atrial fibrillation (ATRIAL_FIBRILLATION = 0), which reduces the mortality probability with an impact of −0.0303, and the presence of ischemic cardiomyopathy (ISCHEMIC_CARDIOMYOPATHY = 1), which surprisingly acts as a protective factor in this model, with an impact of −0.0271. Conversely, the absence of Amiodarone (AMIODARONE = 0) contributes positively to mortality risk, with an impact of 0.0097. The absence of diabetes (DIABETES = 0) provides a protective effect with an impact of −0.0070, while TSH levels between 0.23 and 1.02 slightly increase the risk, with an impact of 0.0031. The presence of CRT (CRT = 1) also reduces the mortality probability, with an impact of −0.0027, indicating a marginal protective role. Other variables, such as a BMI of 18 and the use of ACE inhibitors or ARBs (ACEi/ARBs = 1), exert minor protective effects, with impacts of −0.0010 and −0.0007, respectively. Finally, the presence of an ICD (ICD = 1) and FT4 levels above 1.45 contribute minimally to reducing mortality, each with an impact of −0.0005.

Figure 12, “LIME Explanations for Hospitalization Prediction Model in Hyperthyroid Patients,” provides insights into the model's predictions for hospitalization. Here, the predicted probabilities are also evenly split, with 50% for “YES HOSPITALIZATION” and 50% for “NO HOSPITALIZATION,” indicating no dominant prediction tendency within this patient group.

The absence of atrial fibrillation (ATRIAL_FIBRILLATION = 0) serves as the strongest protective factor, reducing the hospitalization probability with an impact of −0.0309. Similarly, the presence of ischemic cardiomyopathy (ISCHEMIC_CARDIOMYOPATHY = 1) reduces hospitalization likelihood, with an impact of −0.0270. On the other hand, the absence of Amiodarone (AMIODARONE = 0) slightly increases the risk, with an impact of 0.0100. The absence of diabetes (DIABETES = 0) has a protective impact of −0.0076 on hospitalization probability. TSH levels in the range 0.23 < TSH ≤ 1.02 contribute a slight positive influence on hospitalization risk, with an impact of 0.0035. The presence of CRT (CRT = 1) also has a minor protective effect, with an impact of −0.0021, while a BMI of 18 provides additional protection with an impact of −0.0017. Other features exerting limited impacts include LVEF levels within 26.79–30.77, which slightly increase hospitalization likelihood (impact of 0.0005), while the use of ACE inhibitors or ARBs (ACEi/ARBs = 1) adds a minimal positive impact of 0.0004. Age over 70 (AGE >70) serves as a slight protective factor, with an impact of −0.0004, indicating a very marginal influence on hospitalization predictions. These features, though impactful to some extent, play a relatively small role in the overall predictions for mortality and hospitalization in hyperthyroid patients, highlighting the model's balanced treatment of features in predicting outcomes for this group.

### 4.4 Experimental risk stratifications

In this section, we present an experimental approach to risk stratification, where we evaluate and combine the probabilities of mortality and hospitalizations for patients across different thyroid classes and in various optimization scenarios. This approach aims to develop a risk stratification framework that can identify patients at high risk, facilitating targeted interventions. The process utilizes a multi-objective optimization strategy with four scenarios, ultimately visualized in a combined heatmap to summarize risk levels across groups. Our goal is to analyze and combine the risk of Mortality and Hospitalization across four thyroid classes: Euthyroid, Hypothyroid, LT3, and Hyperthyroid. This analysis is performed under four scenarios:

1. Maximize Mortality and Maximize Hospitalization: This scenario identifies conditions that maximize both risks.
2. Maximize Mortality and Minimize Hospitalization: This scenario targets patients with high risk of Mortality but lower risk of Hospitalization.
3. Minimize Mortality and Maximize Hospitalization: This scenario focuses on minimizing Mortality risk while maintaining a higher Hospitalization risk.
4. Minimize Mortality and Minimize Hospitalization: This scenario seeks to minimize both risks, representing the lowest overall risk profile.

Each scenario provides insight into how the balance of Mortality and Hospitalization risks varies across patient classes, highlighting distinct risk profiles for targeted interventions.

To handle these dual objectives—Mortality and Hospitalization—we use a weighted sum approach. This approach is common in multi-objective optimization, where conflicting objectives must be simultaneously optimized. In our context, each objective is calculated based on the probability of Mortality ($p_{Death}$) and the probability of Hospitalization ($p_{Hosp}$), derived from pre-trained ML models. The weighted sum method allows us to combine these objectives into a single metric for easier comparison. The weighted sum method can be represented mathematically as (Equation 16):

(16) CombinedRisk=w1⋅Objective1+w2⋅Objective2

where w1 and w2 are weights for each objective. In this analysis, we have set w1=0.5 and w2=0.5, giving equal importance to both Mortality and Hospitalization. The equal weighting provides a balanced assessment of the risks without favoring one over the other.

The optimization problem is structured around the four scenarios described above. Each scenario is defined by specific objective functions for Mortality and Hospitalization:

• Maximize Mortality & Maximize Hospitalization: $Objective1\;=\;p_{Death}$, $Objective2\;=\;p_{Hosp}$
• Maximize Mortality & Minimize Hospitalization: $Objective1=p_{Death}$, $Objective2\;=\;1-p_{Hosp}$
• Minimize Mortality & Maximize Hospitalization: $Objective1\;=\;1-p_{Death}$, $Objective2=p_{Hosp}$
• Minimize Mortality & Minimize Hospitalization: $Objective1\;=\;1-p_{Death}$, $Objective2\;=\;1-p_{Hosp}$

The predicted probabilities ($p_{Death}$ and $p_{Hosp}$) are derived from pre-trained ML models, such as Random Forest, which estimate the likelihood of Mortality and Hospitalization for each patient. These formulations enable the analysis of specific combinations of high and low risks, tailoring the optimization to address varying clinical priorities and patient profiles. By utilizing these probabilities in the optimization framework, we ensure that the risk stratification process is directly linked to model outputs, providing actionable insights that align with predicted patient outcomes.

The optimization is performed for each thyroid class, and the results are summarized by calculating representative points—average values of Follow-up for Mortality (Mortality_FU) and Follow-up for Hospitalization (Hospi_FU). For each thyroid class and scenario, we compute the mean Hospi_FU and Mortality_FU values, which summarize the overall risk level under the specified conditions. These average values serve as the basis for comparison in the subsequent heatmap analysis. To create a single, interpretable measure of risk, we calculate a Combined Risk Score by averaging the Mortality_FU and Hospi_FU scores, as (Equation 17):

(17) CombinedRisk=w1⋅Death_FU+w2⋅Hospi_FU

where w1=0.5 and w2=0.5. This balanced weighting helps identify thyroid classes and scenarios with higher overall risk, simplifying the complex multi-objective results into a single metric. We assigned equal weights (w1=w2=0.5) to combine mortality and hospitalization risks, ensuring a balanced approach that reflects the clinical importance of both factors. Mortality represents the most severe outcome, while hospitalization significantly impacts quality of life and healthcare costs. By using identical weights, we ensure an unbiased analysis, avoiding distortions and providing an easily interpretable combined risk score. This exploratory approach, aligned with the experimental nature of the study, provides a robust foundation for future research that could explore customized weights based on emerging clinical priorities. Finally, the combined risk is normalized into a percentage for easier interpretation, as (Equation 18):

(18) CombinedRisk(%)=CombinedRiskx100

The final output of this analysis is a heatmap representing the Combined Risk Levels across thyroid classes and scenarios, as shown in Figure 13. Each cell in the heatmap corresponds to a thyroid class-scenario combination, with color intensity indicating the level of combined risk. Darker colors represent higher combined risk scores, highlighting groups with elevated risks for Mortality and/or Hospitalization. The heatmap is generated as follows:

• Data Preparation: The representative points (mean Hospi_FU and Mortality_FU values) are organized into a pivot table with thyroid classes as rows and scenarios as columns. The Combined Risk Score is calculated for each combination.
• Heatmap Visualization: Using *seaborn*, we create a heatmap where each cell is colored according to the Combined Risk Score. Annotations show the exact risk level within each cell, and a color bar to the side provides a legend for interpreting the colors.

The heatmap provides an intuitive visualization of risk distribution across thyroid classes and scenarios:

• High-Risk Cells: Dark red cells indicate thyroid classes and scenarios with higher combined risks. For example, LT3 in the Max-Mortality & Max-Hospitalization scenario shows high risk, suggesting a need for close monitoring in this subgroup.
• Moderate-Risk Cells: Cells with medium color intensity represent scenarios with balanced risks. Hypothyroid and Hyperthyroid classes in the Max-Mortality & Min-Hospitalization and Min-Mortality & Max-Hospitalization scenarios display moderate risk, which may require tailored interventions.
• Low-Risk Cells: Blue cells, particularly in the Min-Mortality & Min-Hospitalization scenario, show the lowest combined risk. These groups may require less intensive follow-up.

The Figure 12 heatmap offers an intuitive visualization of risk distribution, highlighting clear differences between thyroid classes and optimization scenarios. This stratification serves as a basis for personalized clinical decision-making, identifying high-priority groups for intervention.

The analysis of combined risk levels across thyroid classes and scenarios reveals notable variations in risk profiles based on different optimization configurations. For the Euthyroid class, the combined risk is 0.35 when both mortality and hospitalization risks are maximized, indicating that Euthyroid patients exhibit a relatively low level of risk even under high-risk conditions for both factors. When mortality risk is maximized and hospitalization risk minimized, the combined risk rises to 0.50, suggesting a moderate risk level. Similarly, the combined risk remains at 0.50 when mortality risk is minimized and hospitalization risk maximized, indicating that reducing the mortality risk while maintaining high hospitalization risk does not significantly change the overall risk level. Surprisingly, when both risks are minimized, the combined risk increases to 0.65, suggesting that reducing both risks may increase the overall risk profile for Euthyroid patients.

For the Hyperthyroid class, the pattern of combined risk closely mirrors that of the Euthyroid class. With the maximization of both risks, the combined risk is also 0.35, suggesting that Hyperthyroid patients, like Euthyroid patients, maintain a relatively low risk level even under high-risk conditions. When mortality risk is maximized and hospitalization minimized, the combined risk reaches 0.50, a moderate level identical to that of the Euthyroid class. The same combined risk level of 0.50 is observed when mortality risk is minimized and hospitalization maximized. However, when both risks are minimized, the combined risk increases to 0.65, the highest value for this class, indicating a significant rise in overall risk under these conditions.

The Hypothyroid class demonstrates a distinct risk profile. When both mortality and hospitalization risks are maximized, the combined risk reaches 0.58, the highest observed so far, suggesting that for Hypothyroid patients, maximizing both risks considerably increases the overall risk level. In the scenario where mortality risk is maximized and hospitalization minimized, the combined risk reduces to a moderate level of 0.50, which remains unchanged even when mortality risk is minimized and hospitalization risk maximized. However, in a context where both risks are minimized, the combined risk further drops to 0.42, indicating that minimizing both risks has a more pronounced risk-reducing effect for the Hypothyroid class compared to high-risk conditions.

Finally, for the LT3 class, the maximization of both mortality and hospitalization risks results in the highest combined risk of all classes, at 0.63. This finding suggests that LT3 patients are particularly vulnerable in conditions of high mortality and hospitalization risk. When mortality risk is maximized and hospitalization minimized, the combined risk drops to 0.50, representing a moderate risk level consistent with other classes in this scenario. Similarly, when mortality risk is minimized and hospitalization maximized, the combined risk remains stable at 0.50. However, when both risks are minimized, the combined risk falls to the lowest level observed at 0.37, indicating that reducing both risks is associated with a very low overall risk level for the LT3 class.

These findings, illustrated in Figure 4, clearly demonstrate how combined risk levels vary across thyroid classes and scenarios. The Euthyroid and Hyperthyroid classes maintain relatively low risk levels across scenarios, while the Hypothyroid and LT3 classes show greater sensitivity to changes in risk scenarios, with higher combined risk levels in specific configurations of risk maximization or minimization. This analysis provides valuable insights for tailored interventions based on the unique risk profiles of each thyroid class.

### 4.5 Implications, limits and future perspectives

The ML models developed in this study offer significant potential to improve the clinical management of patients with HF and thyroid dysfunctions. By accurately identifying individuals at high risk of mortality and hospitalization, these models enable targeted interventions and personalized treatment strategies. For instance, the early identification of hypothyroid patients with a high likelihood of adverse events could lead to more frequent monitoring, adjustments in pharmacological therapy. Additionally, the interpretation of model outcomes using LIME provides valuable insights to guide clinical decision-making. By highlighting the specific factors contributing to a patient's individual risk, LIME allows clinicians to tailor treatment plans and focus interventions on areas of particular concern.

It is important to acknowledge the limitations of this study to properly interpret the results and guide future research. Although the ML-based approach has shown promising results, the generalizability of the models must be further assessed in larger and more diverse patient populations. The study was retrospective in nature, which introduces potential biases and limits the ability to establish causal relationships. Specifically, there is an inherent risk of selection bias, as patients were not randomly assigned, and the dataset reflects a single-center population with specific inclusion criteria. Information bias and residual confounding may also be present, despite efforts to include a comprehensive set of clinical variables and ensure complete case analysis. Moreover, since the data were not originally collected for predictive modeling purposes, the retrospective design may have introduced selection and information bias. Although only 0.2% of missing values were handled using model-based imputation—which is methodologically appropriate for such low levels of missingness—this approach could still introduce subtle distortions and affect model interpretability, particularly for clinically sensitive variables such as NT-proBNP or thyroid hormones, which may influence risk classification thresholds. These potential biases, related both to the study design and data handling procedures, should be carefully considered when interpreting the results. While the dataset was sizable and well-characterized, these limitations must be considered when interpreting the results. Furthermore, while the statistical analysis included comparisons across multiple variables and subgroups, no formal correction for multiple comparisons was applied. This may increase the risk of Type I error, particularly in exploratory analyses. Therefore, the results should be interpreted with appropriate caution. Future research should incorporate statistical correction techniques—such as Bonferroni or false discovery rate (FDR) adjustments—especially in studies involving formal hypothesis testing across large variable sets. In this study, missing data (accounting for only 0.2% of the dataset) were handled using model-based imputation with a simple decision tree, implemented via the “Impute” widget in Orange. While this approach ensures consistent and reliable estimation of missing values and minimizes information loss, we acknowledge that even low-level imputation may introduce subtle biases or influence model transparency. Future studies should consider comparing multiple imputation techniques to evaluate their impact on the reliability and interpretability of predictive models. Therefore, prospective and multicenter studies with external validation cohorts are strongly recommended to confirm the generalizability and clinical applicability of the proposed models. In this study, the dataset was split into a training set (70%) and a validation set (30%) using the *train_test_split* function from Python's *sklearn* library, with the aim of assessing model performance on unseen internal data and minimizing the risk of overfitting. Additionally, all models were subjected to 10-fold cross-validation to ensure internal consistency and robustness. While these approaches provide strong internal validation, they do not replace the use of independent external datasets. The absence of external validation limits the ability to assess the reproducibility of the model across different populations and healthcare settings. Future research should incorporate external, multicenter cohorts to confirm the generalizability and clinical utility of the proposed framework. Testing the model on broader and more clinically diverse populations will be essential to validate its real-world applicability and ensure its effectiveness in routine clinical practice. Moreover, the lack of prospective validation in the current study represents a significant limitation that further restricts the generalizability of the findings. Although cross-validation and internal testing were rigorously applied, these do not replace the need for validation in real-world, forward-looking clinical environments. Future research should prioritize prospective study designs to verify the model's robustness across diverse patient populations and clinical workflows. While the sample size was substantial, it may not be sufficient to capture the full range of complex interactions between HF and thyroid dysfunctions. Moreover, the demographic composition of the dataset reflects a predominance of male patients (78%), which may introduce gender bias into the model's predictions. This imbalance limits the ability to draw sex-specific conclusions and could impact the model's performance in female subpopulations. Future studies should aim to recruit gender-balanced cohorts to ensure the fairness and representativeness of AI-based risk stratification tools. Additionally, some clinically and socially significant variables—such as medication adherence, health literacy, and socioeconomic status—were not included in the model due to their absence from the structured electronic health records used in this retrospective study. The lack of these variables may limit the completeness and equity of the risk predictions. Future research should prioritize the integration of behavioral and contextual factors to develop more comprehensive and socially aware AI models that better reflect real-world complexities. Further studies in larger, ideally prospective, cohorts would strengthen the study's conclusions and validate its clinical application.

The insights derived from this study pave the way for promising directions in future research. Exploring the integration of additional clinical variables, such as genetic markers and advanced imaging data, could further enhance the predictive accuracy of the models. Incorporating these multidimensional factors could lead to a more comprehensive risk stratification and more precise personalized medicine. Developing ML models capable of predicting not only mortality and hospitalization but also other important patient outcomes, such as quality of life and disease progression, would improve the clinical value of these tools. Additionally, investigating the role of different ML algorithms and optimization techniques could lead to more robust and efficient models. Furthermore, it is essential to study the impact of targeted interventions guided by ML models on patient outcomes. Conducting randomized clinical trials to evaluate the effectiveness of personalized treatment strategies based on model predictions would provide definitive evidence of their clinical benefit. Finally, translating these research findings into practical and accessible clinical tools is essential to realize their full potential. Developing intuitive interfaces and integrating ML models into electronic health record systems would facilitate their widespread adoption and improve patient care. To promote clinical integration, the proposed model could be embedded into electronic health record (EHR) systems as a decision support tool. For example, automatically generated risk scores could trigger alerts for clinicians, prompting earlier intervention or closer monitoring of high-risk patients with thyroid dysfunction and HF. Moreover, the use of interpretable AI techniques such as LIME can help clinicians understand and trust the model's outputs, enhancing transparency and supporting more personalized treatment decisions.

To ensure real-world applicability, future studies should focus on prospective validation using independent and multicenter patient cohorts. This process should involve: (1) recruiting representative populations across different clinical sites; (2) integrating the model into electronic health record systems for real-time risk assessment; (3) comparing clinical decision-making and outcomes with and without model support; and (4) conducting prospective, pragmatic trials to assess the effectiveness of AI-assisted care in routine clinical workflows.

In conclusion, this study demonstrates the immense potential of ML in predicting the risk of mortality and hospitalization in patients with HF and thyroid dysfunctions. AI and ML are increasingly emerging as promising tools to support clinical decision-making and personalize therapeutic pathways, offering new perspectives in the integrated management of cardiovascular and endocrine comorbidities (25). By recognizing the limitations and pursuing future research directions, this field is poised to advance our understanding of this complex interaction and to guide personalized treatment strategies to improve patient outcomes.

## 5 Conclusions

This study highlights ML as a promising tool to enhance risk stratification and treatment personalization for patients with HF and thyroid dysfunctions. Leveraging a comprehensive set of clinical data, the study demonstrates that ML models, particularly the Random Forest algorithm, can accurately predict mortality and hospitalization risk in this patient population.

The good discriminative ability, evidenced by AUC values for mortality prediction (0.797) and hospitalization risk (0.786), underscores the effectiveness of the Random Forest model in distinguishing between high- and low-risk patients. The model's robust performance, evaluated through metrics such as accuracy, precision, recall, and F1 score, further reinforces its reliability for clinical decision support.

Model interpretation using LIME provides valuable insights into the factors contributing to an individual patient's risk. This information enables targeted interventions and personalized treatment strategies, tailored to the specific needs of each patient. For instance, identifying high-risk patients with clinical characteristics, such as the presence of atrial fibrillation or the absence of amiodarone therapy, could lead to more frequent monitoring, adjustments in pharmacological therapy, and careful consideration of interventions such as CRT.

The study analyzed 762 patients, divided into subgroups based on the presence or absence of thyroid dysfunctions. The results revealed significant clinical differences between groups, with LT3 and hypothyroid patients showing a higher risk of atrial fibrillation and elevated levels of NT-proBNP, an indicator of HF severity. These differences underscore the importance of considering thyroid status in risk assessment and treatment planning for patients with HF.

The risk stratification analysis, using a multi-objective optimization strategy, provided additional insights into the risk profiles of different thyroid classes. Hypothyroid and LT3 patients exhibited a higher combined risk in scenarios where both mortality and hospitalization risk were maximized, highlighting their vulnerability under high-risk conditions.

However, the study has certain limitations. Its retrospective nature introduces potential biases, and the generalizability of the findings should be assessed in larger, more diverse patient cohorts. Further prospective studies are needed to validate the study's findings and clinical applicability.

Despite these limitations, the study represents a significant step forward in applying ML to improve care for patients with HF and thyroid dysfunctions. Integrating additional clinical variables, such as genetic markers and advanced imaging data, could further enhance the predictive accuracy of these models. Exploring different ML algorithms and optimization techniques may lead to more robust and efficient models.

In conclusion, this study demonstrates the potential of ML in transforming the management of patients with HF and thyroid dysfunctions. By leveraging ML, clinicians can gain a deeper understanding of individual risk profiles, enabling targeted interventions and personalized treatment strategies to improve patient outcomes and promote more effective healthcare delivery.

## Data Availability

The raw data supporting the conclusions of this article will be made available by the authors, without undue reservation.
